# Protein Biomarker Discovery Studies on Urinary sEV Fractions Separated with UF-SEC for the First Diagnosis and Detection of Recurrence in Bladder Cancer Patients

**DOI:** 10.3390/biom13060932

**Published:** 2023-06-01

**Authors:** Stephanie Jordaens, Eline Oeyen, Hanny Willems, Filip Ameye, Stefan De Wachter, Patrick Pauwels, Inge Mertens

**Affiliations:** 1 Center for Oncological Research (CORE), Integrated Personalized & Precision Oncology Network (IPPON), University of Antwerp, 2610 Wilrijk, Belgium; stephanie.jordaens@uantwerpen.be (S.J.); patrick.pauwels@uza.be (P.P.); 2 Health Unit, Flemish Institute for Technological Research (VITO), 2400 Mol, Belgiumhanny.willems@vito.be (H.W.); 3 Centre for Proteomics (CfP), University of Antwerp, 2020 Antwerp, Belgium; 4 Department of Urology, AZ Maria Middelares, 9000 Ghent, Belgium; filip.ameye@azmmsj.be; 5 Department of Urology, Antwerp University Hospital (UZA), 2650 Edegem, Belgium; stefan.dewachter@uza.be; 6 Laboratory of Pathological Anatomy, Antwerp University Hospital (UZA), 2650 Edegem, Belgium

**Keywords:** liquid biopsy, urine, oncology, non-muscle-invasive bladder cancer (NMIBC), extracellular vesicles, biomarker, proteomics

## Abstract

Urinary extracellular vesicles (EVs) are an attractive source of bladder cancer biomarkers. Here, a protein biomarker discovery study was performed on the protein content of small urinary EVs (sEVs) to identify possible biomarkers for the primary diagnosis and recurrence of non-muscle-invasive bladder cancer (NMIBC). The sEVs were isolated by ultrafiltration (UF) in combination with size-exclusion chromatography (SEC). The first part of the study compared healthy individuals with NMIBC patients with a primary diagnosis. The second part compared tumor-free patients with patients with a recurrent NMIBC diagnosis. The separated sEVs were in the size range of 40 to 200 nm. Based on manually curated high quality mass spectrometry (MS) data, the statistical analysis revealed 69 proteins that were differentially expressed in these sEV fractions of patients with a first bladder cancer tumor vs. an age- and gender-matched healthy control group. When the discriminating power between healthy individuals and first diagnosis patients is taken into account, the biomarkers with the most potential are MASP2, C3, A2M, CHMP2A and NHE-RF1. Additionally, two proteins (HBB and HBA1) were differentially expressed between bladder cancer patients with a recurrent diagnosis vs. tumor-free samples of bladder cancer patients, but their biological relevance is very limited.

## 1. Introduction

Bladder cancer (BC) is the sixth most common cancer in men and seventeenth most common cancer in women worldwide [1]. It is the second most common genitourinary tract malignancy worldwide [2]. BC can be divided into non-muscle-invasive BC (NMIBC) and muscle-invasive BC (MIBC) [3]. NMIBC are cases localized in the mucosa or submucosa, show no invasiveness or metastasis and are usually tumor staged as Ta, T1 or carcinoma in situ (CIS), while MIBC are cases where the tumor has already filtrated the muscle layer [4,5]. Around 70–80% of patients are diagnosed with a non-muscle-invasive tumor and 20–30% with a muscle-invasive tumor [3,6,7]. Patients with NMIBC can undergo curative treatment by transurethral resection. However, MIBC can progress and metastasize rapidly and is usually fatal in the metastatic setting [8]. Low-grade NMIBC patients have a good prognosis with a 5-year survival of 80–90%. Most patients will exhibit at least one recurrence within 5 years with and without progression to MIBC. Currently, follow-up consists of cystoscopy every three to six months for the first two years, followed by less frequent observations in case no recurrences have been detected within this period [3,6]. Early detection of (recurrent) bladder cancer leads to an improved prognosis. 

Currently, NMIBC is diagnosed via cystoscopy or urine cytology. Cystoscopy is not an option for screening purposes, despite providing high diagnostic accuracy, as it is both invasive and painful [9]. On the other hand, urine cytology is non-invasive but shows poor sensitivity, especially for low-stage/low-grade NMIBC [10], making it an inefficient screening test. 

Urine as a liquid biopsy is gaining interest for BC diagnosis. Because urine is non-invasive, it is easy to collect, without the need for a healthcare practitioner. Urine can be collected without limits in volume and is repeatable, allowing for serial sampling and multi-omic analysis. Several urinary biomarkers, such as nuclear matrix protein 22 (NMP22), bladder tumor antigen (BTA), carcinoembryonic antigen and mucins, have been approved by the US Food and Drug Administration. However, they have not been widely adopted due to low specificity and high data heterogeneity [11]. Therefore, non-invasive and more accurate biomarkers are still needed for bladder cancer diagnosis. 

Extracellular vesicles (EVs) are sub-micron (40–1000 nm), membranous vesicles secreted into the extracellular space by many cell types under normal and pathological conditions [12]. EVs play an important role in intercellular communication by regulating a wide range of physiological responses and pathological processes [13]. Cancer cells are known to secrete more EVs than normal cells, and tumor-cell-derived EVs contain cancer-specific components that are capable of promoting cancer progression [14,15]. Urine is expected to contain abundant EVs from bladder cancer tissues, as it comes in direct contact with the urogenital system [16]. The cargo of EVs is thought to reflect the cell type of origin [17,18,19]. In this way, exploring the urinary EV cargo can lead to biomarkers for the diagnosis or prediction of progression of urological diseases [18,20]. Therefore, there is increasing interest in cancer-derived urinary EVs as a promising source of diagnostic biomarkers and therapeutic targets for bladder cancer [18,20,21,22,23,24,25,26,27,28,29]. Consensus has not yet emerged on definitions of EVs. However, the ISEV has published the MISEV guidelines. In these guidelines the ISEV endorses use of the term “small EVs (sEVs)” if the separated vesicles are below 200 nm and if the researchers cannot establish specific markers [30].

Here, a biomarker discovery study was performed using sEVs as sources of protein biomarkers, separated from urine using ultrafiltration (UF) in combination with size-exclusion chromatography (SEC). In the first part of the study, we tried to identify a potential protein biomarker panel for the primary diagnosis of bladder cancer and, more specifically, a potential protein biomarker panel to detect all possible stages and grades of NMIBC [4,5]. The sEV protein contents from urine samples of patients with a first diagnosis of bladder cancer were compared with age- and gender-matched healthy controls. In the second part of the study, the protein profiles of urinary sEVs from patients with a bladder cancer recurrence and patients with no suspicion of a tumor present in the bladder at the moment of urine collection (but with a previous bladder cancer diagnosis) were compared. To generate the protein profiles, we used mass spectrometry, as this technology allows the unbiased identification and quantification of proteins, without the use of antibodies. Because of the size of the cohort in this study, we used a label-free approach, which enables quantification of all identified proteins. Since proteins are identified based on unique peptides, specificity is guaranteed.

## 2. Materials and Methods

### 2.1. Urine Collection

The human biological material (urine) used in this publication was provided by Biobank@UZA (Antwerp, Belgium; ID: BE71030031000) and the Belgian Virtual Tumourbank (Brussel, Belgium), funded by the National Cancer Plan, BBMRI-ERIC [31]. The urine samples were collected at four different locations, i.e., the University Hospital of Antwerp (UZA), General Hospital Maria Middelares (AZMM), General Hospital Turnhout and General Hospital Herentals between 2015 and 2020. Voided urine was obtained with written informed consent (approved by the Ethical Committee of the University of Antwerp and the University Hospital of Antwerp). All samples were collected using the urine vacuette system (Vacuette^®^, Greiner bio-one, Kremsmünster, Austria). The samples were stored immediately at −20 °C. Based upon literature [32], samples were thawed at room temperature and centrifuged at 180× *g* for 10 min at 4 °C and at 1550× *g* for 20 min at 4 °C. The pellets were discarded to prepare cell-free (CF) urine. The samples were subsequently stored at −80 °C until further use. Samples from four experimental groups were collected: primary diagnosis of bladder cancer (n = 50), recurrent diagnosis of bladder cancer (n = 46), tumor free (n = 109) and healthy controls (n = 64). To confirm the absence of a bladder tumor within the healthy control group, a urine cytology and urine analysis with a Multistix 10SG strip-test (Siemens Healthcare, Erlangen, Germany) were performed. The 64 healthy controls included in this study had negative urine cytology and no or mild hematuria (5 samples had mild hematuria: M11, V2, V10, V13, V17).

### 2.2. Extracellular Vesicle (EV) Isolation

Per isolation procedure, 40 to 70 mL of CF urine was thawed and ultrafiltrated using 100 kDa MWCO Centricon^®^ Plus-70 Centrifugal Filter Units (Merck Millipore Ltd., Ireland) to get rid of the solute, smaller particles and proteins. The filtrate was placed on a qEV single 70 nm original column (Izon Science Ltd., Addington, New Zealand). With these columns, 200 µL fractions were collected, starting immediately after placing the sample on the column, with filtered PBS as the elution buffer. The first mL (=dead volume) was collected starting immediately after placing 100 µL filtrate on the column. The next 500 µL was than collected containing the sEVs. The qEV columns were used according to the manufacturer’s instructions.

### 2.3. sEV Characterization

#### 2.3.1. Protein Concentration

The protein concentration was determined using the Micro BCA™ Protein Assay Reagent Kit (Thermo Scientific, Waltham, MA, USA). A standard curve of serially diluted BSA (Thermo Fisher Scientific) in filtered PBS was used. Values were extrapolated from this curve, using a linear equation, with r^2^ > 0.98 for each assay [33].

#### 2.3.2. Transmission Electron Microscopy (TEM)

The quality of EVs was examined using TEM imaging. The sample preparation was adapted from Chen et al. [22]. Briefly, three droplets of the sample were placed on a clean Parafilm, after which a carbon-coated TEM grid was placed on top of the droplets and allowed to stand for 60 min to adsorb the fluid. The grid with adherent EVs was washed three times with PBS for 2 min and 5 times with ultrapure water for 2 min. The droplets were fixed with 2% glutaraldehyde for 10 min and then washed 5 times with ultrapure water for 2 min. The grid was transferred to 2% uranyl acetate and allowed to stand for 15 min. The grid was then incubated in 0.13% methyl cellulose (K5-8) and 0.4% uranyl acetate for 10 min and dried at room temperature before examination with a Tecnai G2 Spirit BioTWIN (FEI, Eindhoven, The Netherlands). All solutions were filtered and the ultrapure water was heated to release the CO_2_.

#### 2.3.3. Nanoparticle Tracking Analysis (NTA)

NTA, using a Zetaview^®^ S/N 18-407 instrument (Particle Metrix GmbH, Inning am Ammersee, Germany), determines the size and concentration of particles ranging from 50 nm to 1 µm in diameter. Samples were diluted 200- to 2000-fold in PBS before measurement. Samples were analyzed in scatter mode with the 488 nm laser and 2 measurements at 11 positions. The temperature was set at 22.0 °C. The shutter was set at 100, sensitivity at 80.0 and frame rate at 30. The number of detected particles in the measurement had to be between 27 and 345, otherwise different dilutions were used to analyze the sEV sample [34]. All analyses were executed using the Zetaview Software (version 8.05.12 SP2).

### 2.4. Proteomic Analysis

#### 2.4.1. Sample Preparation and Liquid Chromatography Followed by Tandem Mass Spectrometry (LC MS/MS)

The same sample preparation protocol as previously described [34] was used for the proteomic analysis. In brief, the sEV fractions were vacuum-dried and lipids and proteins of the sEV fractions were separated by methyl-tert-butyl-ether (MTBE) extraction. The lower hydrophilic fraction was vacuum-dried and used in the proteomic analysis. The vacuum-dried hydrophilic layer was resuspended in 75 µL 5 M urea and the samples were vortexed and sonicated for 10 min. The proteins were reduced in a final concentration of 5 mM dithiothreitol at 60 °C for 30 min and alkylated in a final concentration of 20 mM iodoacetamide in the dark at room temperature for 30 min. Subsequently, 680µL 100mM ammonium bicarbonate was added. For the digestion step, 1 µg trypsin was added per 40 µg of protein and digestion was carried out overnight at 37 °C. Digests were desalted using Pierce C18 spin columns (Thermo Scientific, Waltham, MA, USA) according to the manufacturer’s instructions. The sample preparation of all samples was carried out in 4 different batches on different time points.

For the LC, a reversed phase 200 cm C18 µPAC™ column (Pharmafluidics, Ghent, Belgium) was used. A peptide equivalent to the digest of 1.0 µg of total proteins in 10 µL mobile phase A per sample was prepared to load on this column. A linear gradient of mobile phase B (0.1% formic acid in 98% acetonitrile) from 1 to 45% in 95 min was followed by a steep increase to 90% mobile phase B in 10 min. A steep decrease to 1% mobile phase B was achieved in 5 min and 1% mobile phase B was maintained for 5 min. The flow rate was 400 nL per minute. LC was followed by untargeted shotgun MS/MS and was performed on a Q-Exactive plus MS (Thermo Fisher Scientific). A nanospray ion source (Thermo Fisher Scientific) was used. A full scan spectrum (350 to 1850 *m*/*z*, resolution 70,000, automatic gain control 3 × 106, maximum injection time 100 ms) was followed by a higher-energy collisional dissociation (HCD) tandem MS with a run time of 90 min. Peptide ions were selected for fragmentation as the 10 most intense peaks of a full-scan mass spectrum. HCD scans were acquired in the Orbitrap (resolution 17,500, automatic gain control 1 × 105, maximum injection time 80 ms).

#### 2.4.2. Quality Controls

As previously described [35], the use of an external reference sample through all the analytical LC-MS/MS batches is preferred to have insights into the quality and batch variances of the LC-MS/MS measurements. This sample is expected to have the same intensities of the peptides among the different batches. A pooled sample (=pool) was prepared as a reference sample, containing different samples of all the experimental groups. In Table S1, the sample ID, sample preparation batch, gender of patient and experimental group of the samples that were used are given. Two µg of every sample was used to make the pool (1.5 µg of HC023 was used). Vials containing 1 µg of protein in 10 µL mobile phase A were aliquoted and stored at −80 °C until further analysis.

In the 2 biomarker discovery studies, 4 different experimental groups were present: first diagnosis of bladder cancer, recurrent diagnosis of bladder cancer, tumor free diagnosis with a history of bladder cancer and a healthy control group. To analyze the 269 samples using LC-MS/MS, samples were run randomized and blocked in 16 different analytical LC-MS/MS batches.

#### 2.4.3. Peptide Identification

Proteome Discoverer (PD) software (2.1, Thermo Fisher Scientific) was used to perform database searching against the database containing the Uniprot Human Proteome (ID: UP000005640, downloaded on 25 May 2016), using both Sequest and Mascot search engines. Searches were performed with the following settings: a precursor mass tolerance of 10 ppm and fragment mass tolerance of 0.02 Da. Digestion by trypsin and two missed cleavage sites were allowed. Carbamidomethyl was defined as a fixed modification and phosphorylation (serine, threonine, tyrosine) and oxidation (methionine) were dynamic modifications. The results were filtered with the following parameters: only high-confidence peptides with a global false discovery rate (FDR) < 1% based on a target–decoy approach and first ranked peptides were included in the results.

The MS/MS results (RAW data) together with the PD results were inspected in a quality control (QC) analysis using an in-house-developed QC analysis (set of functions) in R as previously described [35]. Only the samples that passed the QC were used in the biomarker discovery study. Samples that generated low quality data were re-analyzed. If for several reasons samples did not meet the requested QC parameters, for example, reproducibility of retention time and mass calibration, these samples were excluded for further data analysis steps.

#### 2.4.4. Data Enrichment for Peptide Quantification

Before SWISS data enrichment [36] was carried out, mass recalibration and retention time correction were applied, based on the deviations observed in the pool samples. Next, the SWISS tool (an in-house-developed software) was used with the following search and cleaning criteria: The allowed mass difference (∆PPM) was 5 ppm. The complete retention time window where SWISS searched and assessed the peak was 4 min width, 2 min left and 2 min right from the aligned retention time. SWISS searched for the top of the peak in an interval of 1 min. Quantile normalization was performed on the log2 transformed peptide intensities.

### 2.5. Statistical Analysis

The statistical analysis was carried out in R using standard R-core packages [37,38]. An analysis of variance (ANOVA) test was performed per protein, where the unique peptides were considered as a level in the ANOVA. The ANOVA was performed as a pairwise comparison, of which 2 were of interest (healthy vs. first diagnosis NMIBC; tumor free vs. recurrent NMIBC). Afterwards, correction for multiple testing using FDR correction was performed. The protein fold change was calculated as the median fold change of the unique peptides of the protein. Proteins with an absolute median fold change larger than 0.6 and an FDR-corrected p value smaller than 0.05 were considered. Cleaning of this list of differentially expressed proteins was performed based on a manual visual inspection of the RAW data of minimal one unique peptide of these proteins (peak shape, peak tailing/retention time interval, standard deviation of peak intensities and retention times)

### 2.6. Network Analysis

A network analysis was performed using a search tool for the retrieval of interacting genes (STRING) (v11). Interactions in STRING are derived from five main sources: genomic context predictions, high-throughput lab experiments, (conserved) co-expression, automated text-mining and previous knowledge in databases [39,40]. Gene names were entered in the software and Markov clustering (MCL) was performed with the default value (3) of the inflation parameter. Disconnected nodes (up to 3) were hidden and only high-confidence (minimum required interaction score was 0.700) interactions were shown.

### 2.7. EV-TRACK

We have submitted all relevant data of our experiments to the EV-TRACK knowledgebase (EV-TRACK ID: EV230061) [41]. The EV-METRIC score pre-publication was 67%. For the characterization, we refer to our previous paper where the EV isolation method used in this study was described extensively.

## 3. Results

### 3.1. Demographics

Separated sEV fractions of urine samples (n = 269) of individuals (n = 200) were used in these biomarker discovery studies. The samples could be divided into four different experimental groups: Urine samples of age- and gender-matched healthy controls (HC, n = 64) were used with negative urine cytology. Five of the 64 healthy controls (M11, V2, V10, V13, V17) demonstrated mild hematuria symptoms. Urine samples of patients with a first diagnosis of bladder cancer (n = 50) were also collected. In addition, urine samples of patients with a recurrence of bladder cancer (n = 46) and urine samples of bladder cancer patients in follow up with no suspicion of a bladder tumor present at the moment of collection (n = 109) were used. For these last two groups, there were a number of samples derived from the same patient. Twenty patients had 2 urine samples, 14 patients had 3 samples, 4 patients had 4 samples, 3 patients had 5 samples and 1 patient had 6 samples in this biomarker discovery study. Information on demographics of the patients and pathological information, such as stages and tumor grade, of the bladder cancer tumors can be found in Table 1. The tumor grading and staging in Table 1 are from the point in time when the patient was first diagnosed with NMIBC. The gender disproportion in Table 1 is a reflection of the prevalence of bladder cancer in the population. The incidence of bladder cancer is three to four times greater in men than in women. However, principal component analysis demonstrated that gender in our results was not a confounding factor.

### 3.2. sEV Characterization

sEV protein concentration was determined using a Micro BCA™ Protein Assay Reagent Kit (Thermo Scientific, Waltham, MA, USA). The sample preparation protocol was followed using 10 µg of total protein in every sample.

**Figure 1:**
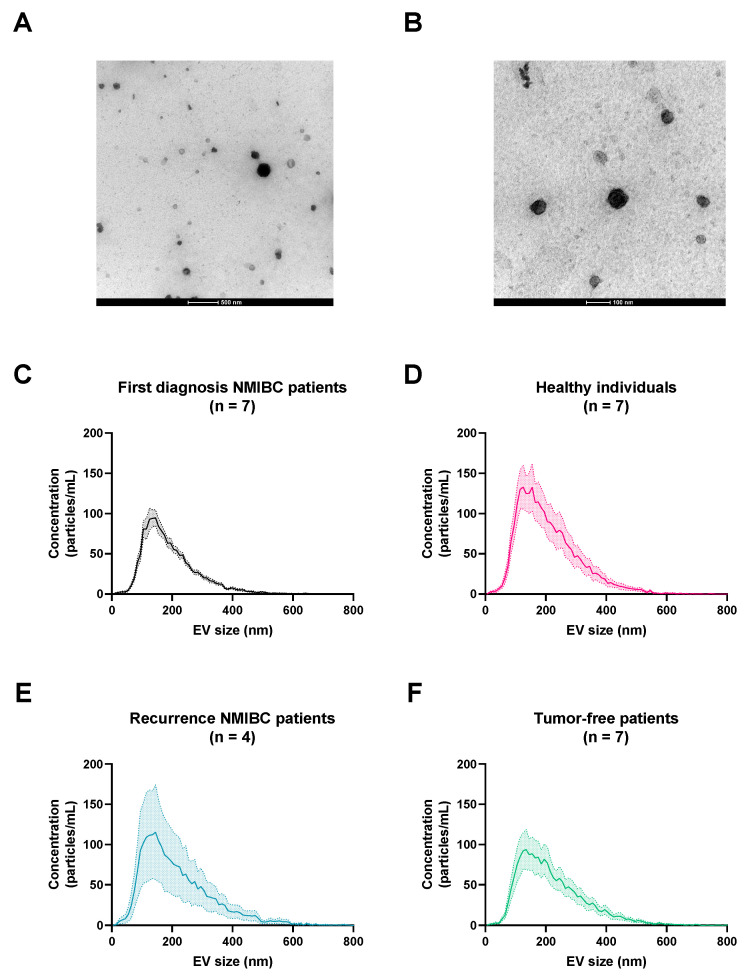
Characterization of urinary sEVs separated with UF-SEC. TEM images of urinary sEVs separated with UF-SEC of a patient with first diagnosis of bladder cancer (BC300) (**A**,**B**). Scale bars are 500 nm (**A**) and 100 nm (**B**). NTA particle size distribution of the four experimental groups: first diagnosis NMIBC patients (**C**), healthy individuals (**D**), recurrence NMIBC patients (**E**), and tumor-free patients (**F**). Abbreviations: NMIBC, non-muscle-invasive bladder cancer; sEV, small extracellular vesicles; TEM, transmission electron microscopy; UF-SEC, ultrafiltration followed by size-exclusion chromatography.

TEM is a commonly used technique to characterize the morphology and size of sEVs. The quality of the urinary sEVs separated with UF-SEC has been previously described [34]. In Figure 1A,B, the TEM images of these urinary sEVs of a bladder cancer patient (BC300) are shown. The separated sEVs were in the size range of 40 to 200 nm. No differences in number, size or morphology of sEVs could be determined using TEM between the four different experimental patient groups (Oeyen E. et al., 2020, unpublished TEM data).

Particle concentration was determined by NTA using a Zetaview^®^ instrument (Particle Metrix) in scatter mode. Of the 4 different experimental groups, 25 samples were analyzed (Figure 1C–F). The number of detected particles in the EV fraction, normalized per mL of starting volume of urine, was between 1.75 × 10^8^ and 7.00 × 10^9^, with an average of 1.64 × 10^9^ particles per mL of starting volume of urine. No correlation was found between the four experimental patient groups concerning the size or concentration of particles, normalized for the starting volume of urine or protein concentration of sEV samples.

### 3.3. Standard Deviation of Patient Samples

**Figure 2:**
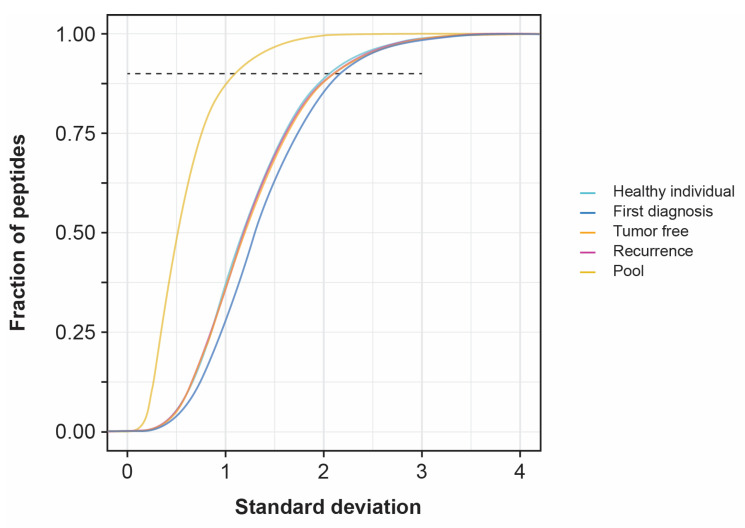
Inter-biological variation of unique peptide intensities. Every line in the graph is the mean of ten replicates. In every replicate, 6 samples were randomly picked over the 16 analytical LC-MS/MS batches. Abbreviations: LC-MS/MS, liquid chromatography followed by tandem mass spectrometry.

The standard deviations of the urinary sEV peptides of healthy individuals have been previously determined [35]. Their ages and genders were related to the bladder cancer population in this biomarker discovery study. This already gave an indication of the sEV peptide variance and the number of samples required to identify statistically relevant biomarkers. However, this outcome needed to be validated in the patient population. The pools in the analytical LC-MS/MS batches of this biomarker discovery study were used to determine the instrumental variation. The variation analysis, using only healthy individuals, was analyzed in one analytical LC-MS/MS batch [35]. For this biomarker discovery study, there is an additional inter-batch effect because of the 16 analytical LC-MS/MS batches. Ten replicates of six randomly selected pools over all the analytical batches were used to determine the standard deviations of the sEV peptides. This approach was also performed to determine the inter-biological variation in the four experimental groups. Figure 2 shows the median curve of these ten replicates per condition (pool, healthy individual, first diagnosis, tumor free, recurrence). For the 90%-least-variable peptides, the standard deviation of the instrumental variation set-up did not exceed 1.1. For the inter-biological variation in the four experimental groups of this biomarker discovery study, the standard deviation was between 2.1 and 2.2. In the previous variation analysis using only healthy individuals, the instrumental standard deviation did not exceed 0.4 for the 90%-least-variable peptides and the inter-biological standard deviation did not exceed 2.0 for the 90%-least-variable peptides [35].

### 3.4. Statistically Differentially Expressed Proteins

Two hypotheses were tested: healthy controls vs. first bladder cancer diagnosis and tumor free vs. recurrence diagnosis. The protein fold change was calculated as the median fold change of the unique peptides of the protein. This resulted in a list of 1226 quantified proteins (Table S2) and, respectively, 193 and 4 proteins that were differentially expressed with an absolute median fold change higher than 0.6 and an FDR-corrected p value lower than 0.05. A careful evaluation of the quality of the proteomic data of these potential biomarkers was performed. The RAW data of at least one unique peptide of the 193 and 4 proteins were manually inspected in every sample. They were checked for peak shape, SWISS score, missing values in all the samples, etc. After this quality control, 69 proteins were kept as statistically relevant differentially expressed in the first biomarker discovery study for the first diagnosis of bladder cancer, and two proteins (HBB and HBA1) were retained as differentially expressed in the biomarker discovery for the detection of bladder cancer recurrence. These detailed lists are shown in Table 2.

### 3.5. Network Analysis

**Figure 3:**
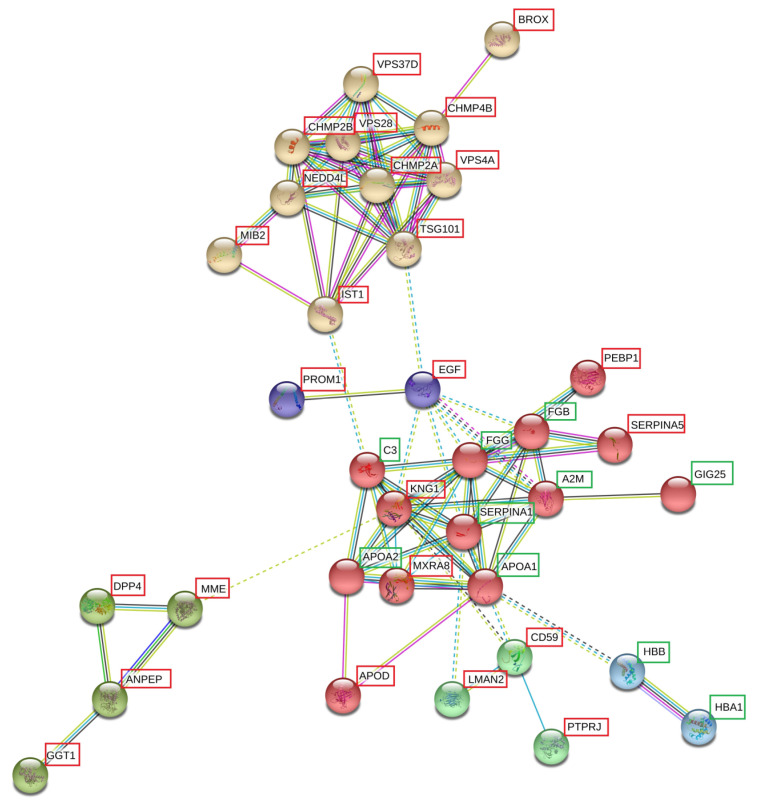
Protein–protein interaction network analysis using STRING (v11) of the 69 differentially expressed proteins for the first detection of bladder cancer. Gene names are indicated. Six networks can be distinguished, indicated in six different colors (brown, purple, light green, red, dark green, and blue). Legend: red, downregulated; green, upregulated.

A network analysis was performed using STRING (v11) with the 69 statistically significant differentially expressed proteins in the first biomarker discovery study for the first bladder cancer diagnosis. The interaction between different proteins is shown and the closer the distance between any 2 proteins in the network, the closer the connection. The connection is based on cited literature and is not based on their interaction in pathways. Six different connected clusters (indicated in six different colors) could be differentiated in this network analysis (Figure 3). All the proteins related to these networks are highlighted in Table 2.

#### 3.5.1. Networks and Pathways

The first network that could be distinguished is the upper brown network (Figure 3), with proteins related to ubiquitination and the ESCRT pathway. Ubiquitination is performed by the ubiquitin proteasome system (UPS) and attaches ubiquitin, a small regulatory protein of 76 amino acids, to proteins, resulting in the post-translational modification of the proteins. The ESCRT dependent pathway for exosome biogenesis requires the sequential function of ESCRT-0, -l, -ll and III complexes. ESCRT-l binds to ubiquitinated cargo proteins and is required for the sorting of endocytic ubiquitinated cargo into multivesicular bodies (MVBs).

The cluster indicated in red was related to fibrinolysis, complement and coagulation cascades (Figure 3). The protein cluster of differentiation 59 (CD59) in the dark green cluster was closely related to the complement cascade (Figure 3). CD59 is an important complement regulatory protein (CRP) that disassembles the membrane attack complex (MAC) of the complement pathway. Different serine protease inhibitors (alpha-2-macroglobulin (A2M), phosphatidyl ethanolamine binding protein 1 (PEBP1), kininogen-1 (KNG1), alpha-1-antitrypsin (SERPINA1), alpha-1-antichymotrypsin (SERPINA3) and plasma serine protease inhibitor (SERPINA5)) were also part of this network and play a role in coagulation. Furthermore, apolipoproteins (APO), APOA1, APOA2 and APOD, linked with coagulation, were detected in this network, whereof the first two were upregulated and APOD was downregulated in this study.

The cluster indicated in blue contained two hemoglobins, hemoglobin subunit alpha (HBA1) and hemoglobin subunit beta (HBB) (Figure 3). HBA1 and HBB were the only statistically significant biomarkers with an absolute fold change larger than 0.6 and an FDR-corrected p value smaller than 0.05 in the other biomarker discovery study with bladder cancer patients with a recurrence diagnosis and tumor-free patients in follow up.

The light green cluster contained different peptidases: aminopeptidase N (ANPEP), dipeptidyl peptidase 4 (DPP4), gamma-glutamyl transpeptidase 1 (GGT1) and neprilysin (MME), which were all downregulated in this study (Figure 3).

Pro-epidermal growth factor (EGF) and prominin-1 (PROM1) were in the purple cluster (Figure 3). In the sEV fractions, PROM1 was downregulated in bladder cancer patients.

#### 3.5.2. Differentially Expressed Proteins Not in Network Analysis

There are still 34 statistically significant expressed proteins with a median absolute fold change larger than 0.6 and an FDR-corrected p value smaller than 0.05 that were not included in the above-mentioned network analysis. The most interesting protein upregulated in the samples of bladder cancer patients was peroxiredoxin 2 (PRDX2). Meanwhile, the most interesting proteins downregulated in the samples of bladder cancer patients were: collagen alpha-1 (VI) chain (COL6A1), fructose biphosphate aldolase B (ALDOB) and mannan-binding lectin serine protease (MASP2). Additionally, three proteins were under-expressed in samples of bladder cancer patients in this study: human leukocyte antigen class II histocompatibility antigen alpha chain (HLA-DMA), Na(+)/H(+) exchange regulatory cofactor 1 (NHE-RF1, SLC9A3R1) and uncharacterized protein C11orf52 (C11orf52).

### 3.6. Discriminating Power of Biomarker Candidates

**Figure 4:**
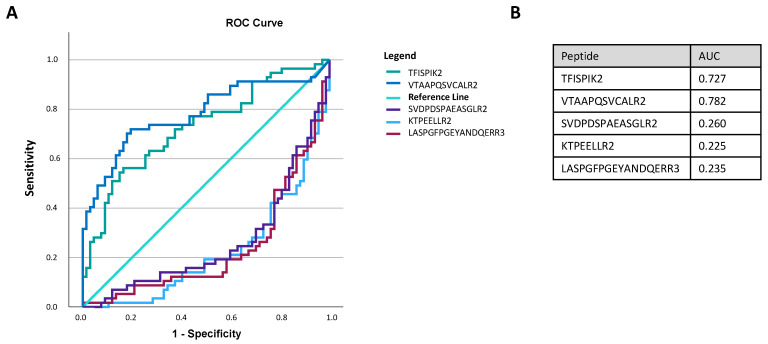
ROC curves and AUC values of potential biomarker candidates. ROC curves (**A**) and AUC values (**B**) of potential biomarker candidates for the first diagnosis of bladder cancer. The peptides above the reference line are discriminative for bladder cancer, the ones below the reference line are discriminative for healthy. The peptide sequences correspond to the following proteins: TFISPIK2 (=C3), VTAAPQSVCALR2 (=A2M), SVDPDSPAEASGLR2 (=NHE-RF1), KTPEELLR2 (=CHMP2A) and LASPGFPGEYANDQERR3 (=MASP2). Abbreviations: A2M, alpha-2-macroglobulin; AUC, area under curve; CHMP2A, charged multivesicular body protein A2; C3, complement C3; MASP2, MBL associated serine protease 2; NHE-RF1, SLC9A3 regulator 1; ROC, receiver operating characteristic.

A good biomarker candidate needs to have a high discriminating power between the diseased and the control group. The distribution of the normalized peak intensities of the unique peptides in the different experimental groups were visualized in ROC curves (Figure 4A). The peptides above the reference line are discriminative for bladder cancer, the ones below the reference line are discriminative for healthy. Based on these ROC curves, LASPGFPGEYANDQERR3 (=MASP2), TFISPIK2 (=C3), VTAAPQSVCALR2 (=A2M), KTPEELLR2 (=CHMP2A) and SVDPDSPAEASGLR2 (=NHE-RF1) have a high discriminating power between the bladder cancer patients and the healthy control group and might be good biomarker candidates.

## 4. Discussion

A biomarker discovery study was performed on the protein content of urinary sEVs, separated with UF-SEC. In the first part, healthy individuals were compared with bladder cancer patients with a primary diagnosis. In the second part, tumor-free patients were compared with patients with a recurrence bladder cancer diagnosis. The separated sEVs were characterized, and, according to the TEM results, they were in the size range of 40 to 200 nm. No correlation could be demonstrated between the numbers of sEVs in the different experimental groups.

The compared experimental groups of these biomarker discovery studies were age and gender balanced (Table 1). This is very important since a subpopulation of the sEV fractions from urine are gender-associated (i.e., prostate-derived sEVs). Furthermore, age influences the senescence of cells and Boulestreau et al. recently demonstrated that mesenchymal-stem-cell-derived EVs are components of the senescence-associated secretory phenotype, i.e., proinflammatory cytokines and chemokines and tissue-damaging proteases and play a critical role in cellular senescence and aging [42]. They suggested that this can have an impact on the molecular cargo of the mesenchymal stem cell derived sEVs from different age groups [42]. Further investigation is needed if the molecular cargo of urinary sEVs is impacted by the age of the patient.

### 4.1. Standard Deviation of Patient Samples and Statistically Differentially Expressed Proteins 

The standard deviation calculation based on only the healthy samples [35] leads to comparable standard deviations for the experimental patient groups used in this discovery study (2.0 vs. 2.1–2.2). The instrumental standard deviation is larger in the biomarker discovery study than in the previous variation analysis on healthy control samples (1.1 vs. 0.6). In the latter one, only one analytical LC-MS/MS batch was performed, thus the batch effects of this biomarker discovery study were not present in this previous variation analysis.

Given the sample size calculation, with the number of samples per experimental group used in this biomarker discovery study, proteins with an absolute fold change of 1.8 could be detected with statistical significance [35]. In this study, this was only the case for the detection of HBA1, HBB and A2M. These are all blood-related proteins and for that reason they are not interesting as potential biomarkers. The sample size of this biomarker discovery study was rather low for a fold change of 0.6, so we have to consider the chance of false positive discoveries and also that real biomarkers might have been missed. In a follow up study, more samples of the experimental groups will have to be included, next to samples coming from patients with other relevant bladder pathologies, to check the specificity of the potential biomarkers. However, to minimize the chance for false positive discoveries, the proteomic RAW data of the 193 differentially expressed proteins with an absolute fold change larger than 0.6 and an FDR-corrected p value smaller than 0.05 underwent a quality check as described in Section 2. This resulted in the refinement of the list into 69 biomarker leads for the first biomarker discovery study. If the protein was identified based on more unique peptides, these proteins were more trusted. Most of the proteins are based on multiple peptides (Table 2). For the ones based on one unique peptide, the proteomic RAW data were determined to be of high quality with almost no missing data (only the unique peptide of HLA-DMA is hydrophilic and suffers from contaminations in the samples resulting in a decreased intensity of this peptide in some pools). The fact that all these potential biomarker leads are based on high quality peptide signals and not noise is promising for their utility as biomarker candidates. 

### 4.2. Network Analysis

#### 4.2.1. Ubiquitination and ESCRT-Pathway-Related Proteins

The first network that could be distinguished is the upper brown network (Figure 3). It contains E3 ligases of the ubiquitin proteasome system (UPS) and components of the ESCRT pathway. 

Ubiquitination is performed by the UPS and attaches ubiquitin, a small regulatory protein of 76 amino acids, to proteins, resulting in the post-translational modification of the proteins. It is a multistep process and requires the sequential action of three enzymes. The first one is the E1 or activation enzyme that recruits free ubiquitin in the cell and activates it in an adenosine triphosphate (ATP)-dependent way. The activated ubiquitin is then transferred to the E2 conjugate enzyme. The E3 ligases are responsible for substrate recognition and facilitates the transfer of ubiquitin to the substrate. The resulting linkage of ubiquitin chains creates a certain topology and dictates the fate of the substrate. For instance, a resulting “closed” or compact conformation leads to degradation of polyubiquitinated proteins, carried out in the 26S macromolecular proteasome complex. In contrast, an “open” conformation results in non-proteasomal signaling functions [43,44]. Two E3 ligases were identified, E3 ubiquitin-protein ligase (MIB2) and E3 ubiquitin protein ligase NEDD4 like (NEDD4L), that were significantly downregulated in the separated sEVs of patients with a first diagnosis of bladder cancer (Table 2). It has been described in the literature that cancer cells exploit the members of the ubiquitination pathway to stabilize aberrant oncogenic signaling, leading to cancer progression and metastasis. Furthermore, downregulation of some E3 ubiquitin ligases has been described as stabilizing aberrant oncogenic signaling [43]. However, the literature is controversial about the role of ubiquitination in cancer and it is not clear what the role of these E3 ubiquitin ligases is in sEVs. 

The ESCRT-dependent pathway for exosome biogenesis requires the sequential function of ESCRT-0, -l, -ll and III complexes. ESCRT-l binds to ubiquitinated cargo proteins and is required for the sorting of endocytic ubiquitinated cargo into multivesicular bodies (MVBs). Vacuolar-protein-sorting-associated protein 28 (VPS28), vacuolar-protein-sorting-associated protein 37D (VPS37D) and tumor susceptibility gene 101 protein (TSG101) are components of the ESCRT-l complex and were significantly downregulated in the sEV fractions of patients with a first bladder cancer diagnosis. Bro 1 containing domain (BROX), charged multivesicular body proteins (CHMP) CHMP2A, CHMP2B, CHMP4B and vacuolar-protein-sorting-associated protein 4A (VPS4A), all components of ESCRT-lll complex, were also significantly downregulated. BROX associates with CHMP4 and plays a role in the sorting of ubiquitinated cargos [45]. The exact role of these ESCRT proteins in sEVs is not clear at the moment. However, TSG101 was also identified as a tumor-suppressor gene and loss of TSG101 is involved in oncogenesis [46,47]. Latosinska et al. (2017) discovered that BROX was also downregulated in bladder cancer tissue samples, according to tumor stage [48]. No conclusion could be made according the number of sEVs produced based on the downregulation of these ESCRT proteins since also vesicles independent of the ESCRT pathway exist. Colombo et al. (2013) concluded that the inhibition of certain ESCRT-l proteins (HRS, STAM1 and TSG101) decreased exosome secretion in cells and inhibition of certain ESCRT-lll proteins (CHMP4C, VPS4B, VTA1, ALIX) increased exosome secretion [49].

#### 4.2.2. Fibrinolysis, Complement Activation and Coagulation Cascades

The cluster indicated in red was related to fibrinolysis, complement and coagulation cascades (Figure 3). The expression of complement C3 (C3) in the sEV fractions of bladder cancer patients was increased in this biomarker discovery study. The upregulation of C3 was also demonstrated in several types of cancer and in different liquid biopsies [50,51,52,53,54]. Increased expression of C3 could indicate the activation of the complement cascade pathway: the immunity system is activated and can destroy tumor cells. This is the fundamental principal of antibody-based immunotherapy: trying to evoke the immune system to eliminate tumor cells [55]. However, complement activation in the tumor microenvironment can also promote tumor growth via overactive complement and chronic inflammation. This promotes tumor immune escape, resulting in tumor progression and metastasis [56,57]. TWIST1 was already known as a biomarker candidate for the detection of bladder cancer [58,59]. It was hypothesized that TWIST1 reduces E-cadherin expression, which is a cell-to-cell adhesion molecule, and loss of its expression results in epithelial-to-mesenchymal transition (EMT), a hallmark of cancer [60]. Cho et al. (2016) showed that TWIST1 also binds to the promotor region of the C3 gene and enhances C3 transcription. They demonstrated that the effect of TWIST1 on E-cadherin is mediated through C3 and C3 decreased E-cadherin expression on cancer cells and promoted epithelial to mesenchymal transition [61]. Therefore, C3 harbors both pro-oncogenic and tumor suppressive functions.

The protein cluster of differentiation 59 (CD59) in the dark green cluster was closely related to the complement cascade (Figure 3). CD59 is an important complement regulatory protein (CRP) that disassembles the membrane attack complex (MAC) of the complement pathway. CD59 is expressed on the surface of tumor cells, potentially in order to limit CDC [62]. However, it has also been shown that the loss of CD59 expression in breast tumors correlates with poor patient survival [63]. CD59 was downregulated in the sEV fractions of bladder cancer patients in this biomarker discovery study. In the study of Abdullah-Soheimi et al. (2010), CD59 was also reduced in the urine of patients with ovarian cancer [64]. There are some possible explanations for the lack of CRPs on EVs: lower CD59 content on the sEVs of bladder cancer patients due to higher CD59 levels on tumor cells or lower CD59 on EVs results in an overactive complement in the tumor microenvironment and this has deleterious effects due to chronic inflammation that promotes carcinogenesis [65]. The true underlying mechanism remains unclear. Vesicular integral membrane protein VIP36 (LMAN2) and protein tyrosine phosphatase receptor type J (PTPRJ) are also nodes in this green cluster. In contrast to this study, LMAN2 was upregulated in the urine of prostate cancer patients [66]. However, PTPRJ was downregulated in the sEV fractions of the bladder cancer patients in this study and is also frequently deleted in several types of cancer [67].

Different serine protease inhibitors (alpha-2-macroglobulin (A2M), phosphatidyl ethanolamine binding protein 1 (PEBP1), kininogen-1 (KNG1), alpha-1-antitrypsin (SERPINA1), alpha-1-antichymotrypsin (SERPINA3), plasma serine protease inhibitor (SERPINA5)) were also part of this network and play a role in coagulation. Coagulation and thrombosis favors tumor progression and metastasis. Again, complement activation has a role in hypercoagulation during cancer [68,69,70]. Tumor cells are surrounded by fibrinogen and can recruit immune cells for the establishment of an inflammatory environment in this fibrin matrix [71]. Two forms of fibrinogen, fibrinogen beta chain (FBB) and fibrinogen gamma chain (FGG), were also upregulated in the samples of bladder cancer patients in this study. Liu et al. (2016) have also shown that an elevated preoperative plasma fibrinogen level is an independent predictor of malignancy and advanced-stage disease in patients with bladder urothelial tumors after investigating 503 patients diagnosed with a first urothelial bladder cancer tumor [72]. The upregulation of fibrinogens in the urine of patients with bladder transitional cell carcinoma was also demonstrated in a study of Li et al. (2011) [73]. Linked to the role of fibrinogen and coagulation in cancer, A2M is also elevated in bladder cancer patients, an inhibitor of fibrinolysis. The upregulation of this protein was also demonstrated by Chen et al. (2012) [22]. In addition, PEBP1, also known as Raf kinase inhibitory protein (RKIP), was downregulated in sEVs of bladder cancer patients in this study. It may act as a metastasis-suppressor gene through the regulation of important signaling cascades, i.e., the RAF-MEK-ERK kinase cascade, G protein-coupled receptors, and the NF-κβ pathway [74,75,76]. As in this biomarker discovery study, downregulated expression of PEBP1 is observed in many cancers as they progress, including bladder cancer [52,77,78,79,80,81]. KNG1 also plays a role in coagulation. KNG1 levels were reduced in the samples of the bladder cancer patients. It was already suggested in literature that KNG1 is an inhibitor of angiogenesis [82]. Furthermore, Abdullah-Soheimi et al. (2010) discovered the significant reduced excretion of KNG1 besides the reduced CD59 levels in urine samples of patients with ovarian carcinoma compared to healthy controls [64]. In addition, several other studies have shown a significant reduction of the KNG1 level in serum and plasma samples in patients with gastrointestinal cancer [83], breast cancer [84] and cervical cancer [85]. However, reduced KNG1 levels may not be cancer-specific since decreased levels have also been reported in the urine of patients with chronic pancreatitis [86], interstitial cystitis [87] and IgA nephropathy [88].

Furthermore, apolipoproteins (APO), APOA1, APOA2 and APOD, linked with coagulation, were detected in this network, whereof the first two were upregulated and APOD was downregulated. The upregulation of APOA1 and APOA2 in the urinary sEVs of bladder cancer patients was also demonstrated in the study of Chen et al. (2013) [89]. APO1 was also upregulated in the urine samples of patients with bladder transitional cell carcinoma [73].

#### 4.2.3. Hemoglobins

The cluster indicated in blue contained two hemoglobins, hemoglobin subunit alpha (HBA1) and hemoglobin subunit beta (HBB) (Figure 3). HBA1 and HBB were the only statistically significant biomarkers with an absolute fold change larger than 0.6 and an FDR-corrected p value smaller than 0.05 in the other biomarker discovery study with bladder cancer patients with a recurrence diagnosis and tumor-free patients in follow up. The upregulation of these proteins in samples of patients with a bladder cancer tumor can be linked with the contamination of blood due to hematuria, indicating that these proteins might not be tumor-specific.

#### 4.2.4. Peptidases

The light green cluster contained different peptidases: aminopeptidase N (ANPEP), dipeptidyl peptidase 4 (DPP4), gamma-glutamyl transpeptidase 1 (GGT1) and neprilysin (MME), which were all downregulated in this study (Figure 3). The literature describes that DPP4 act as a tumor suppressor gene and was downregulated in some types of cancer [90,91,92,93]. In addition, the downregulated expression of ANPEP has also been observed in different types of carcinomas [52,94,95]. MME is also known as CD10. Both loss of expression of MME [96,97,98] and higher expression levels of MME are reported [99,100], varying by tissue type and disease state. In this biomarker discovery study, a down regulation was observed in bladder cancer patients. GGT1 was part of the glutathione pathway and glutathione has a crucial role in the removal and detoxification of carcinogens. Alterations in this pathway can have a profound effect on cell survival [101].

#### 4.2.5. Pro-Epidermal Growth Factor (EGF) and Prominin-1 (PROM1)

EGF and PROM1 were in the purple cluster (Figure 3). PROM1 was overexpressed in different types of tumors [102]. In the sEV fractions, PROM1 was downregulated in bladder cancer patients. The biological role of PROM1 remains unclear [103]. Another proteomic biomarker discovery study in abundant-protein-depleted urine revealed the under expression of pro-epidermal growth factor (EGF) in the bladder cancer patient group vs. hernia patients [89]. Chen et al. (2013) demonstrated previously that urinary EGF levels were not significantly altered in patients with kidney cancer, rendering them a useful biomarker for bladder cancer [89].

#### 4.2.6. Differentially Expressed Proteins Not in Network Analysis

There are still 34 statistically significant expressed proteins with a median absolute fold change larger than 0.6 and an FDR-corrected p value smaller than 0.05 that were not included in the above-mentioned network analysis. The most interesting proteins are discussed below. 

Peroxiredoxin 2 (PRDX2) was upregulated in the samples of the bladder cancer patients. This was also demonstrated in a study of Chen et al. (2010), where the potential of PRDX2 as a stage discriminator for bladder cancer was well demonstrated [104]. Collagen alpha-1(VI) chain (COL6A1) was downregulated in the bladder cancer group in this biomarker discovery study. The collagen proteins play a role in maintaining the integrity of various tissues and COL6A1 was also downregulated in prostate cancer [105], colorectal cancer [106] and ovarian cancer [107]. Fructose bisphosphate aldolase B (ALDOB) was downregulated in the sEV fractions of bladder cancer patients in this study. Downregulation of ALDOB has also been observed in gastric cancer patients and this was associated with poor prognosis [108]. By contrast, ALDOB overexpression was observed during the epithelial to mesenchymal transition in colorectal adenocarcinoma in a study of Li et al. (2017) [109]. The role of ALDOB in cancer is still controversial and more exploration is mandatory. Human leukocyte antigen class II histocompatibility antigen alpha chain (HLA-DMA) was also under-expressed in this biomarker discovery study. However, caution should be taken with this candidate biomarker since the identification and quantification of this protein was based on one hydrophilic peptide. It has been suggested that the under expression of HLA-DMA had an effect on the persistent binding of CLIP during the HLA class II antigen presenting, resulting in a lower CD4+ T cell response [110]. This is in accordance with the findings of Filipazzi et al. (2012) that tumor-derived sEVs prevent T-cell activation [111]. The downregulation of HLA-DMA has also been previously seen in head and neck squamous cell carcinoma [112]. Another under-expressed protein in the bladder cancer patient group of this study was Na(+)/H(+) exchange regulatory cofactor 1 (NHE-RF1, SLC9A3R1) and it has been previously demonstrated that NHE-RF1 is a tumor suppressor [113,114]. In a study of Georgescu et al. (2016), NHE-RF1 deficiency in mice resulted in increased tumor burden [113].

Upregulation of the wnt-β-catenin pathway was demonstrated in NHE-RF1-deficient tumors. In normal conditions, NHE-RF1 interacts with β-catenin and suppresses the wnt-β-catenin pathway [113]. Downregulation of NHE-RF1 increased this pathway. NHE-RF1 downregulation has also been demonstrated previously in colon cancer [115]. It has also been demonstrated that in cervical cancer, NHE-RF1 was down regulated in cells that were resistant for cisplatin-based chemotherapy [116]. Furthermore, an uncharacterized protein C11orf52 (C11orf52) was under-expressed in the sEV fractions of the bladder cancer patients. This protein was previously identified in sEVs separated from urine [117]. In a recent study of Hwang et al. (2020), it was suggested by bioinformatic prediction that this uncharacterized protein might play a role in the wnt signaling pathway [118]. In addition, mannan-binding lectin serine protease (MASP2), a serum protease that plays an important role in the activation of the complement system via mannose-binding lectin, was downregulated in the sEV fractions of the bladder cancer patients.

### 4.3. Potential Biomarkers

The majority of the identified biomarker candidates have been previously described in the literature. A lot of these proteins were identified in studies exploring plasma, serum or cell-free urine. This raises the question whether the identified proteins are really sEV-derived or are contaminants of the sEV fractions due to the sEV separation method used. It is possible that some proteins are soluble proteins in liquid biopsies and are also located in sEVs or associated with sEVs. For example NHE-RF1, MME and C3 were also identified in the EV cargo of a study of Gupta et al. [119]. The question again is whether these sEV fractions using differential UC are pure vesicles or if these proteins are also contaminants in the isolates. Ultimately, the origin of the biomarker needs to be checked before a diagnostic test could be developed. Therefore, techniques to separate sEVs from urine with high purity are necessary. Furthermore, bladder cancer cell lines can be used in combination with high-purity sEV separation methods to avoid highly abundant soluble urinary proteins in the sEV isolates. In a recent study of Dhondt et al., bottom-up Optiprep density gradient centrifugation was demonstrated to separate sEVs and soluble proteins from urine with high specificity and repeatability [120]. Using this separation method, they detected 3686 and 1996 proteins in an “EV-enriched” and “protein-enriched” fraction, respectively. A list of 684 proteins was identified as putative contaminants of urinary sEV isolates, based on their selective identification in the “protein-enriched” fraction only [120]. In our list of 69 biomarker candidates for both biomarker discovery studies, no candidate was part of this list of putative contaminants. The study of Dhondt et al. (2020) demonstrated that a bottom-up Optiprep density gradient results in the separation of more pure EV-fractions compared to our UF + SEC method, resulting in increased protein identifications (3686 compared to 1226) [120]. However, this method has a lower recovery efficiency (30% vs. 60%) and is time consuming (>21 h), which is impractical when performing large biomarker discovery studies [94]. Exploring the proteomics data of both studies, 17 biomarker candidates in our study were solely identified in their “EV-enriched” fraction and not in their “protein-enriched” fraction, indicating that they are probably not identified as urinary soluble proteins.

In the second biomarker discovery study to detect a biomarker panel to monitor bladder cancer patients in follow-up, only HBB and HBA1 were identified as statistically significant biomarkers with an absolute fold change larger than 0.6 and an FDR-corrected p value smaller than 0.05. The anti-cancer therapies that these patients received, influence the tumor microenvironment and impact the EV release and urinary sEV content of the secreted EVs by tumor cells in response to the therapy [121,122,123]. In addition, sEVs might also be responsible for enhanced prometastatic capacity and mediate resistance after treatment [124,125,126,127,128]. This complicates the detection of biomarker candidates for the detection of a recurrence, independent of the previous anti-cancer treatments the patient received. Further research is required to evaluate the impact of each therapy on urinary sEVs. Also worth mentioning is that 46 urine samples of the bladder cancer patients with a recurrence diagnosis were used, which were derived from only 34 different patients. For the patients in follow-up with no suspicion of a bladder tumor present at the moment of urine collection, 109 urine samples were derived from only 52 different patients. In addition, nine patients had both a tumor free and a recurrence sample in this biomarker discovery study, complicating the statistical analysis since the two groups include partially paired data.

It remains questionable whether apolipoproteins or proteins involved in the complement and coagulation cascade can be highly specific markers for bladder cancer. However, most studies describe no effect of hematuria on the biomarker levels of apolipoproteins [129]. In addition, complement biomarkers are detected in a wide variety of pathological conditions [130,131]. 

In the result section, the identified biomarker candidates were discussed in detail. Comparing this data set to a previous review, combining all known candidate biomarkers [11], POSTN, H2B1K, HEXB, CD36, 5T4, CD73 and EDIL3 were not identified in this proteomic data set. Some proteins such as CD44, MUC4, GPRC5A, BSG, RETN, EPS8L2, EPS8L1, EHD4, ITIH2, APOB, CA1, S100A4 and TACSTD2 were identified. This could be the result of the sample size in this biomarker discovery or the fact that the conclusion in the previously described studies were based on extremely low sample sizes. In addition, since different subsets of sEVs are obtained with different separation methods, this will result in different outcomes. Furthermore, an important parameter is the heterogeneity of the bladder tumors. The stage and grade of the bladder tumor can determine the molecular cargo of the sEVs and thus influence the outcome in biomarker discovery studies.

Other groups have been investigating urinary EVs and EV proteins for bladder cancer detection and monitoring [26,27,28,29]. One study showed that although patients were histologically tumor-free after cystectomy, the bladder urine contained exosomes with a carcinogenic metabolic profile. This suggests a continuous release of exosomes from the bladder, which may promote recurrence at distant sites through metabolic rewiring, even after apparent complete removal of the tumor [26]. Another study demonstrated that most of the proteins identified in tissue-exudative EVs are also present in urinary EVs [27]. The authors combined proteomic analysis of urinary EVs and tissue-exudative EVs to identify urinary EV biomarker proteins for the detection of bladder cancer. They identified heat-shock protein 90, syndecan-1 and myristoylated alanine-rich C-kinase substrate as BC-specific EV proteins representing both potential biomarkers and therapeutic targets. However, their strategy is time consuming and requires expensive equipment, which limits its use beyond a scientific research setting [27].

### 4.4. Future Research

In the field of cancer proteomics or oncoproteomics, comparing the proteome of samples by quantitative proteomics from different conditions, i.e., tumor vs. healthy, can result in the identification of differentially expressed proteins due to abnormal functioning of proteins in cancer conditions [132]. This will provide insights into the underlying mechanisms of carcinogenesis, cancer progression and metastasis. The presence or abundance of certain biomarkers can be used for the diagnosis, monitoring or therapy of cancer [133]. In this study, a proteomic-based biomarker discovery study was performed to identify biomarker candidates for the diagnosis and monitoring of bladder cancer patients. Therefore, mass spectrometry (MS) was used to investigate the potential of urinary sEV-derived proteins as biomarkers for bladder cancer.

MS is a commonly used technology to analyze sensitively unknown proteins. This is advantageous over other proteomic techniques such as Western blot, immunohistochemistry and enzyme-linked immune sorbent assay (ELISA), that only can identify and quantify known proteins because antibodies are required for their detection [134]. Antibody-based methods of protein detection are highly specific but have limitations. Their progress has been hampered by barriers in reproducibility, standardization, small patient cohorts, inability to multiplex assays and the high cost and manual labor time to develop validated ELISA assays [135,136]. MS has emerged as a promising platform to overcome several of these limitations and implement high-throughput proteomics. This is because MS can be highly accurate and reproducible and can measure multiple analytes simultaneously [135,136]. In this study, we have identified some proteins as potential biomarkers for the diagnosis and monitoring of bladder cancer. However, these results need to be confirmed using targeted proteomics methods such as immunoassays, ELISA or LC-MS/MS before being clinically applicable.

## 5. Conclusions

This pilot protein biomarker discovery study on urinary sEV fractions using UF SEC identified 69 protein biomarker candidates for the first diagnosis of bladder cancer, based on high quality proteomic data. This means that the proteins were identified and quantified based on more than one peptide and the raw data were manually controlled. Some biologically relevant biomarker candidates were detected such as GGT1, PEBP1, PTPRJ, PRDX2, NHE-RF1, C11orf52 and MASP2. Regarding the discriminating power between the two groups of some potential biomarkers, MASP2, C3, A2M, CHMP2A and NHE-RF1 were identified as promising biomarker candidates. Moreover, a final independent validation study is required including a validation of these biomarker candidates with a negative control group with urogenital pathologies, to eliminate non-specific biomarkers for the detection of bladder cancer, based on a targeted protein quantification, like targeted MS or antibody-based technologies.

## Figures and Tables

**Table 1:** Demographics and bladder cancer tumor types.

	Healthy Controls	First Diagnosis of Bladder Cancer	Diagnosis of Bladder Cancer Relapse	Tumor Free
**X number of samples**	64	50	46	109
**Derived from X number of patients**	64	50	34	52
**Age (years)**				
Average	69.2	71.9	73.0	70.7
Minimum	40	42	48	35
Maximum	83	89	91	89
**Sex N (%)**				
Male	39 (61%)	39 (78%)	29 (85%)	37 (71%)
Female	25 (39%)	11 (22%)	5 (15%)	15 (29%)
Tumor stage *				
Ta	NA	15	25	NA
T1 (including Tis)	NA	30	19	NA
T2	NA	5	2	NA
Tumor grade *				
1	NA	1	18	NA
2	NA	24	8	NA
3	NA	25	20	NA
**Hospital**				
UZA	32	5	25	17
AZMM	0	39	9	34
AZ Herentals	0	6	0	1
AZ Turnhout	32	0	0	0

* Note: When the determination of the tumor stage and/or tumor grade posed difficulties, the samples were categorized in this table as the worst case scenario.

**Table 2:** Proteins statistically differentially expressed in biomarker discovery studies. The proteins are given for the comparison between healthy controls and first diagnosis NMIBC cases and for the comparison of tumor-free and recurrence NMIBC cases. The proteins highlighted in green are the proteins for the first diagnosis of bladder cancer found in the network analysis using STRING (v11). Abbreviations: FDR, false discovery rate; NMIBC, non-muscle-invasive bladder cancer.

Protein Name	Gene Name	Number of Unique Peptides (seq. z)	Median Fold Change	FDR-Corrected *p* Value
**Healthy controls vs. first diagnosis NMIBC**				
Hemoglobin subunit alpha	HBA1	6	3.31	1.01 × 10^−6^
Hemoglobin subunit beta	HBB	9	3.21	2.76 × 10^−5^
Alpha-2-macroglobulin	A2M	35	2.14	3.03 × 10^−15^
Fibrinogen gamma chain	FGG	18	1.64	4.38 × 10^−4^
Fibrinogen beta chain	FGB	24	1.63	3.48 × 10^−7^
Apolipoprotein A2	APOA2	10	1.40	2.19 × 10^−4^
Complement C3	C3	65	1.27	2.08 × 10^−21^
Apolipoprotein A1	APOA1	29	1.25	1.12 × 10^−11^
T-complex protein 1 subunit eta	CCT7	1	1.11	2.33 × 10^−3^
Peroxiredoxin-2	PRDX2	13	0.98	2.09 × 10^−4^
Histone H4	HIST1H4A	6	0.95	5.57 × 10^−4^
Alpha-1-antitrypsin	SERPINA1	24	0.82	6.40 × 10^−5^
Alpha-1-antichymotrypsin	SERPINA3/GIG25	15	0.63	1.32 × 10^−4^
Apolipoprotein D	APOD	14	−0.62	1.74 × 10^−3^
Tumor susceptibility gene 101 protein	TSG101	16	−0.63	1.56 × 10^−2^
Prominin-1	PROM1	32	−0.64	1.73 × 10^−4^
Galectin-3-binding protein	LGALS3BP	19	−0.64	5.10 × 10^−5^
Annexin A11	ANXA11	35	−0.65	1.45 × 10^−7^
Neprilysin	MME	28	−0.67	1.32 × 10^−4^
Vacuolar-protein-sorting-associated protein 4A	VSP4A	13	−0.67	6.04 × 10^−4^
Phospholipid scramblase 1	PLSCR1	7	−0.73	3.94 × 10^−2^
Vacuolar-protein-sorting-associated protein 37D	VPS37D	11	−0.75	2.80 × 10^−4^
Aminopeptidase N	ANPEP	36	−0.76	1.44 × 10^−9^
Solute carrier family 12 member 1	SLC12A1	26	−0.76	1.32 × 10^−4^
Sodium channel protein type 4 subunit alpha	SCN4A	1	−0.76	3.96 × 10^−2^
E3 ubiquitin-protein ligase	MIB2	1	−0.78	1.64 × 10^−2^
MARCKS-related protein	MARCKSL1	1	−0.79	2.19 × 10^−2^
Cluster of differentiation 59	CD59	14	−0.80	1.83 × 10^−6^
Aminoacylase-1	ACY1	6	−0.80	3.92 × 10^−2^
Plasma serine protease inhibitor	SERPINA5	10	−0.81	2.90 × 10^−3^
Vacuolar-protein-sorting-associated protein 28	VPS28	4	−0.82	3.06 × 10^−2^
Stomatin-like protein 3	STOML3	1	−0.82	1.26 × 10^−2^
Coiled-coil domain-containing protein 168	CCDC168	1	−0.83	4.38 × 10^−3^
Dipeptidase 1	DPEP1	12	−0.83	3.85 × 10^−2^
Prostatic acid phosphatase	ACPP	12	−0.83	2.60 × 10^−3^
Basal cell adhesion molecule	BCAM	11	−0.84	1.27 × 10^−2^
Na(+)/H(+) exchange regulatory cofactor NHE-RF1	NHE-RF1/SLC9A3R1	11	−0.85	3.98 × 10^−3^
Mucin-1	MUC1	11	−0.87	4.44 × 10^−3^
Lipopolysaccharide-induced tumor necrosis factor-alpha factor	LITAF	1	−0.90	5.03 × 10^−3^
Dipeptidyl peptidase 4	DPP4	14	−0.91	3.54 × 10^−3^
Ubiquitin-fold modifier-conjugating enzyme 1	UFC1	1	−0.91	2.05 × 10^−3^
BRO1 domain-containing protein	BROX	15	−0.96	1.01 × 10^−4^
Protein tyrosine phosphatase receptor type J	PTPRJ	16	−0.96	6.71 × 10^−5^
Charged multivesicular body protein 4b	CHMP4B	9	−0.97	7.67 × 10^−4^
Kininogen-1	KNG1	17	−0.97	4.92 × 10^−6^
Vasorin	VASN	20	−0.97	6.57 × 10^−5^
IST1 homolog	IST1	18	−0.98	2.67 × 10^−4^
Pro-epidermal growth factor	EGF	24	−1.00	2.22 × 10^−7^
Leucine-rich repeat-containing protein 71	LRRC71	1	−1.03	2.78 × 10^−3^
Phosphatidylethanolamine-binding protein 1	PEBP1	17	−1.06	1.40 × 10^−4^
Charged multivesicular body protein 2b	CHMP2B	6	−1.08	3.01 × 10^−3^
Vesicular integral membrane protein VIP36	LMAN2	13	−1.09	2.22 × 10^−4^
Uncharacterized protein C11orf52	C11orf52	7	−1.09	3.42 × 10^−2^
G-protein coupled receptor 98	GPR98	1	−1.11	5.27 × 10^−3^
Lysophosphatidylcholine acyltransferase 2	LPCAT2	1	−1.11	4.66 × 10^−3^
Charged multivesicular body protein 2a	CHMP2A	7	−1.12	7.27 × 10^−3^
Ubiquitin carboxyl-terminal hydrolase	UCHL1	1	−1.12	1.34 × 10^−3^
Mannosyl-oligosaccharide 1.2-alpha-mannosidase IA	MAN1A1	6	−1.16	3.68 × 10^−2^
E3 ubiquitin-protein ligase NEDD4 like	NEDD4L	1	−1.18	2.95 × 10^−3^
HLA class II histocompatibility antigen. DM alpha chain	HLA-DMA	1	−1.21	2.78 × 10^−3^
Collagen alpha-1(VI) chain	COL6A1	18	−1.27	2.34 × 10^−5^
Mannan-binding lectin serine protease 2	MASP2	10	−1.29	3.59 × 10^−3^
Cytochrome b reductase 1	CYBRD1	1	−1.30	1.17 × 10^−3^
Semaphorin-3G	SEMA3G	1	−1.32	2.49 × 10^−3^
Small integral membrane protein 5	SMIM5	1	−1.33	1.26 × 10^−3^
Fructose-bisphosphate aldolase B	ALDOB	9	−1.42	4.89 × 10^−3^
Uromodulin	UMOD	40	−1.43	1.21 × 10^−12^
Matrix-remodeling-associated protein 8	MXRA8	12	−1.45	4.58 × 10^−5^
Gamma-glytamyl transpeptidase 1	GGT1	1	−1.80	2.15 × 10^−4^
**Tumor-free vs. recurrence NMBIC**				
Hemoglobin subunit alpha	HBA1	6	1.10	3.19 × 10^−3^
Hemoglobin subunit beta	HBB	9	0.86	3.39 × 10^−2^

## Data Availability

The data that support the findings of this study are available in this article and the Supplementary Materials. Further data are available from the corresponding author upon reasonable request.

## Supplementary Materials

The following supporting information can be downloaded at: https://www.mdpi.com/article/10.3390/biom13060932/s1, Table S1: Patient samples used for pool; Table S2: The full list of 1266 quantified proteins.

## Abbreviations

ANOVA	Analysis of variance
ATP	Adenosine triphosphate
BC	Bladder cancer
cf	Cell-free
EMT	Epithelial-to-mesenchymal transition
EV	Extracellular vesicle
FDR	False discovery rate
HC	Healthy control
HCD	Higher-energy collisional dissociation
LC-MS/MS	Liquid chromatography followed by tandem mass spectrometry
MCL	Markov clustering
MIBC	Muscle-invasive bladder cancer
MTBE	Methyl-tert-butyl-ether
MVBs	multivesicular bodies
NMIBC	Non-muscle-invasive bladder cancer
NTA	Nanoparticle tracking analysis
PD	Proteome discoverer
ppm	Parts per million
QC	Quality control
SEC	Size-exclusion chromatography
sEV	Small extracellular vesicles
STRING	Search tool for the retrieval of interacting genes
TEM	Transmission electron microscopy
UF	Ultrafiltration
UPS	Ubiquitin proteasome system
