# Supplementary material for: Protein Biomarker Discovery Studies on Urinary sEV Fractions Separated with UF-SEC for the First Diagnosis and Detection of Recurrence in Bladder Cancer Patients
Source: Biomolecules. 2023 Jun 1;13(6):932. doi: 10.3390/biom13060932 (PMC10296596; doi:10.3390/biom13060932)
Supplement: Supplementary file 1 [file biomolecules-13-00932-s001.zip › biomolecules-2384391-supplementary.pdf]

Supplementary Materials

Table S1: Patient samples used for pool - Abbreviations: BC, bladder cancer.

Sample ID	Sample preparation batch	Gender	Experimental group
HC010	1	Female	Healthy
HC004	1	Male	Healthy
M5	2	Male	Healthy
V3	2	Female	Healthy
M12	2	Male	Healthy
M1	2	Male	Healthy
M17	2	Male	Healthy
HC023	2	Male	Healthy
HC003	1	Male	Healthy
BC118	1	Female	First BC diagnosis
BC156	1	Male	First BC diagnosis
BC301_1	4	Male	First BC diagnosis
BC402	2	Female	First BC diagnosis
BC400	2	Male	First BC diagnosis
BC309	2	Male	First BC diagnosis
BC803	2	Male	First BC diagnosis
BC129	2	Male	First BC diagnosis
BC154	2	Male	First BC diagnosis
BC130_1	4	Male	Tumor free with history of BC
BC015_4	3	Male	Tumor free with history of BC
BC119_1	4	Male	Tumor free with history of BC
BC156_3	4	Male	Tumor free with history of BC
BC052_3	3	Male	Tumor free with history of BC
BC126_1	3	Female	Tumor free with history of BC
BC040_1_1930	3	Male	Tumor free with history of BC
BC012_2	3	Male	Tumor free with history of BC
BC808_1	3	Male	Tumor free with history of BC
BC301_2	2	Male	Tumor free with history of BC
BC014_1	4	Male	Relapse of BC
BC011_2	3	Male	Relapse of BC
BC046_1	3	Male	Relapse of BC
BC184_1	3	Female	Relapse of BC
BC143_1_2080	3	Male	Relapse of BC
BC806_1	3	Male	Relapse of BC
BC102_1	3	Male	Relapse of BC
BC140_1_2022	4	Male	Relapse of BC

Table S2: The full list of 1,226 quantified proteins.

Protein name	Gene name	Number of unique peptides (seq, z)
1,5-anhydro-D-fructose reductase	AKR1E2	1
14 kDa phosphohistidine phosphatase	PHPT1	2
14-3-3 protein beta/alpha	YWHAB	8
14-3-3 protein epsilon	YWHAE	2
14-3-3 protein eta	YWHAH	2
14-3-3 protein gamma	YWHAG	2
14-3-3 protein sigma	SFN	3
14-3-3 protein theta	YWHAQ	5
14-3-3 protein zeta/delta	YWHAZ	12
15-hydroxyprostaglandin dehydrogenase [NAD(+)]	HPGD	6
1-phosphatidylinositol 4,5-bisphosphate phosphodiesterase beta-3	PLCB3	1
1-phosphatidylinositol 4,5-bisphosphate phosphodiesterase delta-1	PLCD1	12
2',3'-cyclic-nucleotide 3'-phosphodiesterase	CNP	15
2'-deoxynucleoside 5'-phosphate N-hydrolase 1	DNPH1	1
3 beta-hydroxysteroid dehydrogenase/Delta 5-->4-isomerase type 1	HSD3B1	1
3-hydroxybutyrate dehydrogenase type 2	BDH2	4
3-mercaptopyruvate sulfurtransferase	MPST	8
40S ribosomal protein S24	RPS24	1
4F2 cell-surface antigen heavy chain	SLC3A2	5
4-trimethylaminobutyraldehyde dehydrogenase	ALDH9A1	3
5'(3')-deoxyribonucleotidase, cytosolic type	NT5C	2
60S acidic ribosomal protein P1	RPLP1	1
60S ribosomal protein L12	RPL12	1
60S ribosomal protein L7	RPL7	1
6-phosphogluconate dehydrogenase, decarboxylating	PGD	7
6-phosphogluconolactonase	PGLS	14
ABHD14A-ACY1 readthrough	ABHD14A-ACY1	1
Abl interactor 1	ABI1	1
Abl interactor 1	ABI1	2
Acetyl-CoA acetyltransferase, cytosolic	ACAT2	1
Acid ceramidase	ASAH1	4
Actin-related protein 2	ACTR2	4
Actin-related protein 2/3 complex subunit 1A	ARPC1A	1
Actin-related protein 2/3 complex subunit 1B	ARPC1B	3
Actin-related protein 2/3 complex subunit 2	ARPC2	3
Actin-related protein 2/3 complex subunit 4	ARPC4	3
Actin-related protein 2/3 complex subunit 5	ARPC5	1
Actin-related protein 3	ACTR3	8
Acylamino-acid-releasing enzyme	APEH	1

Protein name	Gene name	Number of unique peptides (seq, z)
Acyl-CoA synthetase short-chain family member 3, mitochondrial	ACSS3	1
Acyl-CoA-binding protein	DBI	1
Acyl-protein thioesterase 1	LYPLA1	3
Adapter molecule crk	CRK	2
Adenine phosphoribosyltransferase	APRT	3
Adenosylhomocysteinase	AHCY	4
Adenylate kinase isoenzyme 1	AK1	2
Adenylate kinase isoenzyme 1	AK1	2
Adenylyl cyclase-associated protein 1	CAP1	1
Adipogenesis regulatory factor	ADIRF	5
ADP-ribosyl cyclase/cyclic ADP-ribose hydrolase 2	BST1	1
ADP-ribosyl cyclase/cyclic ADP-ribose hydrolase 2	BST1	2
ADP-ribosylation factor 4	ARF4	1
ADP-ribosylation factor 6	ARF6	4
ADP-ribosylation factor-like protein 13B	ARL13B	1
ADP-ribosylation factor-like protein 15	ARL15	3
ADP-ribosylation factor-like protein 15	ARL15	5
ADP-ribosylation factor-like protein 3	ARL3	1
Adseverin	SCIN	8
Aflatoxin B1 aldehyde reductase member 3	AKR7A3	3
Aflatoxin B1 aldehyde reductase member 4	AKR7L	1
Aggrecan core protein	ACAN	6
Aggrecan core protein	ACAN	6
Agrin	AGRN	7
Alanine--tRNA ligase, cytoplasmic	AARS	1
Alcohol dehydrogenase [NADP(+)]	AKR1A1	7
Alcohol dehydrogenase class-3	ADH5	6
Aldehyde dehydrogenase family 3 member B1	ALDH3B1	2
Aldo-keto reductase family 1 member C2	AKR1C2	1
Aldose 1-epimerase	GALM	2
Aldose reductase	AKR1B1	7
Alpha/beta hydrolase domain-containing protein 14B	ABHD14B	6
Alpha/beta hydrolase domain-containing protein 17C	ABHD17C	1
Alpha-1-acid glycoprotein 1	ORM1	2
Alpha-1-antichymotrypsin	SERPINA3	15
Alpha-1-antitrypsin	SERPINA1	20
Alpha-1-antitrypsin	SERPINA1	24
Alpha-1B-glycoprotein	A1BG	26
Alpha-2-antiplasmin	SERPINF2	3
Alpha-2-HS-glycoprotein	AHSG	1
Alpha-2-HS-glycoprotein	AHSG	19
Alpha-2-macroglobulin	A2M	35
Alpha-2-macroglobulin-like protein 1	A2ML1	1

Protein name	Gene name	Number of unique peptides (seq, z)
Alpha-actinin-1	ACTN1	1
Alpha-actinin-4	ACTN4	9
Alpha-aminoadipic semialdehyde dehydrogenase	ALDH7A1	2
Alpha-amylase 1	AMY1A	1
Alpha-centractin	ACTR1A	1
Alpha-enolase	ENO1	29
Alpha-N-acetylgalactosaminidase	NAGA	1
Alpha-N-acetylglucosaminidase	NAGLU	24
Alpha-parvin	PARVA	1
Amiloride-sensitive amine oxidase [copper-containing]	AOC1	4
Aminoacylase-1	ACY1	6
Aminopeptidase B	RNPEP	3
Aminopeptidase N	ANPEP	36
Ammonium transporter Rh type C	RHCG	2
Angiopoietin-related protein 2	ANGPTL2	8
Angiotensin-converting enzyme 2	ACE2	5
Angiotensin-converting enzyme	ACE	6
Angiotensinogen	AGT	8
Ankyrin repeat domain-containing protein 24	ANKRD24	1
Annexin A1	ANXA1	22
Annexin A11	ANXA11	35
Annexin A2	ANXA2	7
Annexin A3	ANXA3	8
Annexin A4	ANXA4	15
Annexin A5	ANXA5	20
Annexin A6	ANXA6	8
Annexin A7	ANXA7	8
Annexin A9	ANXA9	6
Anoctamin-6	ANO6	3
Anthrax toxin receptor 1	ANTXR1	1
Antithrombin-III	SERPINC1	1
Apolipoprotein A-I	APOA1	29
Apolipoprotein A-II	APOA2	10
Apolipoprotein A-II	APOA2	3
Apolipoprotein A-IV	APOA4	33
Apolipoprotein B-100	APOB	16
Apolipoprotein C-I	APOC1	1
Apolipoprotein D (Fragment)	APOD	2
Apolipoprotein D	APOD	14
Apolipoprotein E	APOE	14
Aquaporin-1	AQP1	3
Aquaporin-2	AQP2	8
Arginase-1	ARG1	3
Argininosuccinate synthase	ASS1	16

Protein name	Gene name	Number of unique peptides (seq, z)
Aromatic-L-amino-acid decarboxylase	DDC	2
Arrestin domain-containing protein 1	ARRDC1	5
Arylsulfatase A	ARSA	3
Aspartate aminotransferase, cytoplasmic	GOT1	7
ATP synthase subunit alpha, mitochondrial	ATP5A1	1
ATP-citrate synthase	ACLY	1
ATP-sensitive inward rectifier potassium channel 1	KCNJ1	2
Attractin	ATRN	2
Azurocidin	AZU1	2
Band 3 anion transport protein	SLC4A1	3
Barrier-to-autointegration factor	BANF1	1
Basal cell adhesion molecule	BCAM	11
Basement membrane-specific heparan sulfate proteoglycan core protein	HSPG2	15
Basigin	BSG	2
Beta-1,4-galactosyltransferase 1	B4GALT1	4
Beta-1,4-glucuronyltransferase 1	B4GAT1	3
Beta-2-glycoprotein 1	APOH	1
Beta-2-microglobulin	B2M	1
Beta-arrestin-1	ARRB1	1
Beta-enolase	ENO3	2
Beta-galactosidase	GLB1	5
Betaine--homocysteine S-methyltransferase 1	BHMT	8
Bifunctional epoxide hydrolase 2	EPHX2	1
Bifunctional purine biosynthesis protein PURH	ATIC	2
Biglycan	BGN	3
Bile salt-activated lipase	CEL	2
Biotinidase	BTD	1
Bisphosphoglycerate mutase	BPGM	2
Bone marrow stromal antigen 2	BST2	1
Bone morphogenetic protein 7	BMP7	1
BPI fold-containing family B member 2	BPIFB2	2
Brain acid soluble protein 1	BASP1	4
Brain-specific angiogenesis inhibitor 1-associated protein 2	BAIAP2	4
Brain-specific angiogenesis inhibitor 1-associated protein 2	BAIAP2	13
Brain-specific angiogenesis inhibitor 1-associated protein 2-like protein 1	BAIAP2L1	11
Breakpoint cluster region protein	BCR	2
Breast carcinoma-amplified sequence 1	BCAS1	3
BRO1 domain-containing protein BROX	BROX	15
BTB/POZ domain-containing protein KCTD12	KCTD12	1
Cadherin-15	CDH15	7
Cadherin-16	CDH16	1
Calbindin	CALB1	5

Protein name	Gene name	Number of unique peptides (seq, z)
Calcineurin B homologous protein 1	CHP1	1
Calcium and integrin-binding protein 1	CIB1	6
Calcium-binding protein 39	CAB39	4
Calcium-binding protein 39-like	CAB39L	2
Calcium-independent phospholipase A2-gamma	PNPLA8	1
Calcyphosin	CAPS	1
Calcyphosin	CAPS	8
Calmodulin-like protein 5	CALML5	1
Calpain small subunit 1	CAPNS1	3
Calpain-1 catalytic subunit	CAPN1	6
Calpain-2 catalytic subunit	CAPN2	2
Calpain-5	CAPN5	1
Calpain-5	CAPN5	18
Calpain-7	CAPN7	21
cAMP-dependent protein kinase catalytic subunit alpha	PRKACA	2
cAMP-dependent protein kinase catalytic subunit beta	PRKACB	1
cAMP-dependent protein kinase type I-alpha regulatory subunit	PRKAR1A	1
cAMP-dependent protein kinase type II-alpha regulatory subunit	PRKAR2A	4
Capping protein (Actin filament) muscle Z-line, beta, isoform CRA_d	CAPZB	1
Carbonic anhydrase 1	CA1	9
Carbonic anhydrase 2	CA2	15
Carbonic anhydrase 4	CA4	8
Carbonyl reductase [NADPH] 1	CBR1	11
Carboxymethylenebutenolidase homolog	CMBL	3
Carboxypeptidase B2	CPB2	1
Carboxypeptidase M	CPM	6
Carboxypeptidase N subunit 2	CPN2	3
Carboxypeptidase Q	CPQ	1
Carboxy-terminal domain RNA polymerase II polypeptide A small phosphatase 1 (Fragment)	CTDSP1	1
Carboxy-terminal domain RNA polymerase II polypeptide A small phosphatase 1	CTDSP1	1
Carcinoembryonic antigen-related cell adhesion molecule 5	CEACAM5	2
Carcinoembryonic antigen-related cell adhesion molecule 5	CEACAM5	4
Carcinoembryonic antigen-related cell adhesion molecule 7	CEACAM7	3
Cartilage intermediate layer protein 1	CILP	1
Cartilage intermediate layer protein 2	CILP2	4
Cartilage oligomeric matrix protein	COMP	1
Casein kinase I isoform gamma-1	CSNK1G1	1
Caspase-14	CASP14	4
Catalase	CAT	4
Catechol O-methyltransferase	COMT	6

Protein name	Gene name	Number of unique peptides (seq, z)
Cathelicidin antimicrobial peptide	CAMP	1
Cathepsin B	CTSB	3
Cathepsin D	CTSD	10
Cathepsin G	CTSG	3
Cathepsin Z	CTSZ	3
CD177 antigen	CD177	2
CD2-associated protein	CD2AP	16
CD44 antigen	CD44	1
CD59 glycoprotein	CD59	4
CD59 glycoprotein	CD59	14
CD63 antigen	CD63	1
CD81 antigen	CD81	3
CD9 antigen	CD9	6
cDNA FLJ55673, highly similar to Complement factor B (EC 3.4.21.47)	N/A	7
Cell cycle control protein 50A	TMEM30A	1
Cell division control protein 42 homolog	CDC42	12
Cell division cycle 5-like protein	CDC5L	1
Cell surface glycoprotein MUC18	MCAM	2
Cellular retinoic acid-binding protein 2	CRABP2	2
Centromere protein T	CENPT	1
Cerebral cavernous malformations 2 protein	CCM2	1
Ceruloplasmin	CP	23
Ceruloplasmin	CP	28
Charged multivesicular body protein 1a	CHMP1A	5
Charged multivesicular body protein 1b	CHMP1B	4
Charged multivesicular body protein 2a	CHMP2A	7
Charged multivesicular body protein 2b	CHMP2B	6
Charged multivesicular body protein 3	CHMP3	4
Charged multivesicular body protein 4a	CHMP4A	2
Charged multivesicular body protein 4b	CHMP4B	9
Charged multivesicular body protein 4c	CHMP4C	2
Charged multivesicular body protein 5	CHMP5	10
Charged multivesicular body protein 6	CHMP6	2
Chloride intracellular channel protein 1	CLIC1	16
Chloride intracellular channel protein 3	CLIC3	4
Chloride intracellular channel protein 4	CLIC4	1
Chloride intracellular channel protein 6	CLIC6	4
Cholesteryl ester transfer protein	CETP	1
Choline transporter-like protein 1	SLC44A1	1
Choline transporter-like protein 2	SLC44A2	12
Choline transporter-like protein 4	SLC44A4	1
Chondroitin sulfate proteoglycan 4	CSPG4	3
Cingulin	CGN	1

Protein name	Gene name	Number of unique peptides (seq, z)
C-Jun-amino-terminal kinase-interacting protein 4	SPAG9	1
Clathrin heavy chain 1	CLTC	3
Clusterin	CLU	17
Coactosin-like protein	COTL1	4
Cofilin-1	CFL1	1
Cofilin-1	CFL1	7
Cofilin-2	CFL2	3
Coiled-coil and C2 domain-containing protein 1A	CC2D1A	6
Coiled-coil domain-containing protein 168	CCDC168	1
Collagen alpha-1(III) chain	COL3A1	1
Collagen alpha-1(VI) chain	COL6A1	18
Collagen alpha-1(XV) chain	COL15A1	2
Collagen alpha-1(XVIII) chain	COL18A1	2
Collagen alpha-2(I) chain	COL1A2	1
Collagen alpha-2(IV) chain	COL4A2	3
Collagen alpha-3(VI) chain	COL6A3	5
Complement C1q subcomponent subunit B	C1QB	1
Complement C1q subcomponent subunit C	C1QC	2
Complement C1r subcomponent-like protein	C1RL	3
Complement C1s subcomponent	C1S	1
Complement C2	C2	1
Complement C3	C3	65
Complement C4-A	C4A	1
Complement C4-A	C4A	1
Complement C4-A	C4A	1
Complement C4-B	C4B	2
Complement C4-B	C4B	2
Complement C5	C5	3
Complement component C7	C7	1
Complement component C8 gamma chain	C8G	1
Complement component C9	C9	4
Complement decay-accelerating factor	CD55	6
Complement factor B	CFB	8
Complement factor D	CFD	2
Complement factor I	CFI	1
Complement receptor type 1	CR1	8
Constitutive coactivator of PPAR-gamma-like protein 2	FAM120C	2
Copine-1	CPNE1	1
Copine-2	CPNE2	1
Copine-3	CPNE3	13
Copine-5	CPNE5	1
Copine-8	CPNE8	8
Corneodesmosin	CDSN	1
Cornifelin	CNFN	1

Protein name	Gene name	Number of unique peptides (seq, z)
Cornifin-A	SPRR1A	1
Coronin-1A	CORO1A	2
Coronin-1B	CORO1B	3
Corticosteroid-binding globulin	SERPINA6	1
Creatine kinase B-type	CKB	5
CTD small phosphatase-like protein	CTDSPL	2
Cubilin	CUBN	21
Cullin-associated NEDD8-dissociated protein 1	CAND1	1
Cyclin-Y-like protein 1	CCNYL1	1
Cystatin-A	CSTA	1
Cystatin-B	CSTB	3
Cystatin-C	CST3	2
Cysteine and glycine-rich protein 1	CSRP1	2
Cysteine-rich and transmembrane domain-containing protein 1	CYSTM1	1
Cysteine-rich C-terminal protein 1	CRCT1	1
Cysteine-rich motor neuron 1 protein	CRIM1	3
Cysteine-rich protein 2	CRIP2	4
Cysteine-rich tail protein 1	CYSRT1	4
Cysteine-rich tail protein 1	CYSRT1	1
Cytochrome b reductase 1	CYBRD1	1
Cytochrome c oxidase subunit 6B2	COX6B2	1
Cytochrome P450 26B1	CYP26B1	1
Cytoplasmic aconitate hydratase	ACO1	3
Cytoplasmic dynein 2 heavy chain 1	DYNC2H1	1
Cytoplasmic FMR1-interacting protein 1	CYFIP1	1
Cytoplasmic FMR1-interacting protein 2	CYFIP2	1
Cytoplasmic tRNA 2-thiolation protein 2	CTU2	1
Cytosol aminopeptidase	LAP3	2
Cytosolic 10-formyltetrahydrofolate dehydrogenase	ALDH1L1	1
Cytosolic non-specific dipeptidase	CNDP2	14
D-3-phosphoglycerate dehydrogenase	PHGDH	6
DCC-interacting protein 13-beta	APPL2	1
DDB1- and CUL4-associated factor 11	DCAF11	1
D-dopachrome decarboxylase	DDT	1
D-dopachrome decarboxylase	DDT	1
Dehydrogenase/reductase SDR family member 2, mitochondrial	DHRS2	1
Deleted in malignant brain tumors 1 protein	DMBT1	5
Delta-aminolevulinic acid dehydratase	ALAD	1
DENN domain-containing protein 1A	DENND1A	1
Deoxyribonuclease-1	DNASE1	3
Deoxyribonuclease-2-alpha	DNASE2	1
Dermatopontin	DPT	1

Protein name	Gene name	Number of unique peptides (seq, z)
Dermcidin	DCD	4
Desmocollin-1	DSC1	2
Desmoglein-1	DSG1	4
Desmoplakin	DSP	23
Destrin	DSTN	6
Dihydropteridine reductase	QDPR	1
Dihydropyrimidinase	DPYS	1
Dipeptidase 1	DPEP1	12
Dipeptidyl peptidase 1	CTSC	2
Dipeptidyl peptidase 2	DPP7	4
Dipeptidyl peptidase 4	DPP4	14
Disabled homolog 2	DAB2	1
Disco-interacting protein 2 homolog B	DIP2B	3
Disintegrin and metalloproteinase domain-containing protein 9	ADAM9	2
DnaJ homolog subfamily A member 1	DNAJA1	1
DnaJ homolog subfamily A member 2	DNAJA2	1
DnaJ homolog subfamily B member 2	DNAJB2	1
Drebrin-like protein	DBNL	2
Dual specificity protein phosphatase 3	DUSP3	2
Dynamin-2	DNM2	3
Dynamin-3	DNM3	1
Dynein heavy chain 7, axonemal	DNAH7	1
Dystroglycan	DAG1	2
E3 ubiquitin-protein ligase Itchy homolog	ITCH	6
E3 ubiquitin-protein ligase MIB2	MIB2	1
E3 ubiquitin-protein ligase NEDD4-like	NEDD4L	1
E3 ubiquitin-protein ligase NEDD4-like	NEDD4L	1
E3 ubiquitin-protein ligase pellino homolog 2	PELI2	1
E3 ubiquitin-protein ligase TRIM17	TRIM17	1
Echinoderm microtubule-associated protein-like 2	EML2	1
Echinoderm microtubule-associated protein-like 2	EML2	1
Ectonucleotide pyrophosphatase/phosphodiesterase family member 6	ENPP6	1
EGF-containing fibulin-like extracellular matrix protein 1	EFEMP1	4
EH domain-containing protein 1	EHD1	1
EH domain-containing protein 1	EHD1	12
EH domain-containing protein 3	EHD3	2
EH domain-containing protein 4	EHD4	18
Elongation factor 2	EEF2	9
Endonuclease domain-containing 1 protein	ENDOD1	4
Endosialin	CD248	7
Envoplakin	EVPL	47
Eosinophil cationic protein	RNASE3	2

Protein name	Gene name	Number of unique peptides (seq, z)
Eosinophil peroxidase	EPX	1
Ephrin type-B receptor 4	EPHB4	3
Ephrin type-B receptor 6	EPHB6	2
Ephrin-B1	EFNB1	4
Epidermal growth factor receptor kinase substrate 8 (Fragment)	EPS8	1
Epidermal growth factor receptor kinase substrate 8	EPS8	23
Epidermal growth factor receptor kinase substrate 8-like protein 1	EPS8L1	20
Epidermal growth factor receptor kinase substrate 8-like protein 2	EPS8L2	33
Epithelial discoidin domain-containing receptor 1	DDR1	1
ER membrane protein complex subunit 7	EMC7	1
Erythrocyte band 7 integral membrane protein	STOM	9
Ester hydrolase C11orf54	C11orf54	4
Eukaryotic translation initiation factor 6	EIF6	4
Excitatory amino acid transporter 3	SLC1A1	2
Exportin-2	CSE1L	1
Extracellular superoxide dismutase [Cu-Zn]	SOD3	3
Ezrin	EZR	41
F-actin-capping protein subunit alpha-1	CAPZA1	2
F-actin-capping protein subunit alpha-2	CAPZA2	3
F-actin-capping protein subunit beta	CAPZB	1
F-actin-uncapping protein LRRC16A	CARMIL1	1
Far upstream element-binding protein 2	KHSRP	1
Far upstream element-binding protein 2	KHSRP	1
Fas apoptotic inhibitory molecule 1	FAIM	1
Fatty acid synthase	FASN	3
Fatty acid-binding protein, epidermal	FABP5	2
Fatty acid-binding protein, heart	FABP3	2
F-box only protein 50	NCCRP1	1
FERM and PDZ domain-containing protein 1	FRMPD1	1
Ferritin heavy chain	FTH1	2
Ferritin light chain	FTL	1
Fibrinogen alpha chain	FGA	24
Fibrinogen beta chain	FGB	24
Fibrinogen gamma chain	FGG	1
Fibrinogen gamma chain	FGG	18
Fibroblast growth factor 1	FGF1	1
Fibroleukin	FGL2	2
Fibronectin	FN1	1
Fibulin-1	FBLN1	3
Fibulin-1	FBLN1	4
Filaggrin-2	FLG2	3

Protein name	Gene name	Number of unique peptides (seq, z)
Filamin-B	FLNB	2
Flavin reductase (NADPH)	BLVRB	1
Flotillin-1	FLOT1	11
Flotillin-2	FLOT2	2
Folate receptor alpha	FOLR1	3
Formimidoyltransferase-cyclodeaminase	FTCD	3
Fructose-1,6-bisphosphatase 1	FBP1	8
Fructose-bisphosphate aldolase A	ALDOA	13
Fructose-bisphosphate aldolase B	ALDOB	9
Fructose-bisphosphate aldolase C	ALDOC	3
Fumarylacetoacetase	FAH	2
Galectin-1	LGALS1	3
Galectin-12	LGALS12	1
Galectin-3	LGALS3	6
Galectin-3-binding protein	LGALS3BP	19
Galectin-7	LGALS7	4
Galectin-8	LGALS8	1
Gamma-glutamyl hydrolase	GGH	3
Gamma-glutamylaminecyclotransferase	GGACT	1
Gamma-glutamylcyclotransferase	GGCT	1
Gamma-glutamyltransferase 6	GGT6	4
Gamma-glutamyltranspeptidase 1	GGT1	1
Gamma-soluble NSF attachment protein	NAPG	2
Gamma-synuclein	SNCG	4
Ganglioside GM2 activator	GM2A	3
Gap junction alpha-3 protein	GJA3	1
GDP-L-fucose synthase	TSTA3	1
GDP-mannose 4,6 dehydratase	GMDS	1
Gelsolin	GSN	12
Glucose-6-phosphate isomerase	GPI	4
Glutaminyl-peptide cyclotransferase	QPCT	1
Glutamyl aminopeptidase	ENPEP	18
Glutaredoxin-1	GLRX	1
Glutathione peroxidase 1	GPX1	1
Glutathione reductase, mitochondrial	GSR	3
Glutathione S-transferase Mu 2	GSTM2	1
Glutathione S-transferase Mu 3	GSTM3	7
Glutathione S-transferase omega-1	GSTO1	2
Glutathione S-transferase P	GSTP1	6
Glutathione S-transferase theta-1	GSTT1	2
Glutathione synthetase	GSS	1
Glyceraldehyde-3-phosphate dehydrogenase	GAPDH	16
Glyceraldehyde-3-phosphate dehydrogenase, testis-specific	GAPDHS	1

Protein name	Gene name	Number of unique peptides (seq, z)
Glycerol-3-phosphate dehydrogenase [NAD(+)], cytoplasmic	GPD1	1
Glycerol-3-phosphate dehydrogenase 1-like protein	GPD1L	3
Glycine--tRNA ligase	GARS	1
Glycogen phosphorylase, brain form	PYGB	1
Glyoxalase domain-containing protein 4	GLOD4	6
Glyoxylate reductase/hydroxypyruvate reductase	GRHPR	3
Glyoxylate reductase/hydroxypyruvate reductase	GRHPR	1
Golgi-associated plant pathogenesis-related protein 1	GLIPR2	3
G-protein coupled receptor 98	GPR98	1
G-protein coupled receptor family C group 5 member B	GPRC5B	4
G-protein coupled receptor family C group 5 member C	GPRC5C	3
Grancalcin	GCA	2
Granulins	GRN	2
Group XV phospholipase A2	PLA2G15	2
Growth factor receptor-bound protein 2	GRB2	3
Growth factor receptor-bound protein 7	GRB7	2
Growth/differentiation factor 15	GDF15	3
GTPase HRas	HRAS	1
GTPase KRas	KRAS	1
GTPase NRas	NRAS	1
GTP-binding nuclear protein Ran (Fragment)	RAN	2
GTP-binding nuclear protein Ran	RAN	2
GTP-binding protein Di-Ras2	DIRAS2	1
Guanine nucleotide-binding protein G(i) subunit alpha-1	GNAI1	8
Guanine nucleotide-binding protein G(i) subunit alpha-2	GNAI2	14
Guanine nucleotide-binding protein G(I)/G(S)/G(O) subunit gamma-10	GNG10	1
Guanine nucleotide-binding protein G(I)/G(S)/G(O) subunit gamma-12	GNG12	3
Guanine nucleotide-binding protein G(I)/G(S)/G(O) subunit gamma-5	GNG5	2
Guanine nucleotide-binding protein G(I)/G(S)/G(O) subunit gamma-7	GNG7	1
Guanine nucleotide-binding protein G(I)/G(S)/G(T) subunit beta-1	GNB1	10
Guanine nucleotide-binding protein G(I)/G(S)/G(T) subunit beta-2	GNB2	7
Guanine nucleotide-binding protein G(k) subunit alpha	GNAI3	8
Guanine nucleotide-binding protein G(q) subunit alpha	GNAQ	8
Guanine nucleotide-binding protein subunit alpha-11	GNA11	6
Guanine nucleotide-binding protein subunit alpha-13	GNA13	10
Guanine nucleotide-binding protein subunit alpha-14	GNA14	2
Guanine nucleotide-binding protein subunit beta-4	GNB4	1
Haptoglobin	HP	7

Protein name	Gene name	Number of unique peptides (seq, z)
HCG2002594, isoform CRA_c	SEPT5	1
Heat shock 70 kDa protein 12A	HSPA12A	2
Heat shock 70 kDa protein 4	HSPA4	1
Heat shock cognate 71 kDa protein	HSPA8	13
Heat shock protein 75 kDa, mitochondrial	TRAP1	1
Heat shock protein beta-1	HSPB1	9
Heat shock protein HSP 90-alpha	HSP90AA1	3
Heat shock protein HSP 90-beta	HSP90AB1	1
Heat shock-related 70 kDa protein 2	HSPA2	6
Heme-binding protein 1	HEBP1	4
Hemicentin-1	HMCN1	4
Hemoglobin subunit alpha	HBA1	6
Hemoglobin subunit beta (Fragment)	HBB	3
Hemoglobin subunit beta	HBB	9
Hemoglobin subunit delta (Fragment)	HBD	1
Hemoglobin subunit delta	HBD	3
Hemopexin	HPX	9
Heparin cofactor 2	SERPIND1	3
Hepatocyte growth factor-regulated tyrosine kinase substrate	HGS	1
High affinity cAMP-specific and IBMX-insensitive 3',5'-cyclic phosphodiesterase 8A	PDE8A	1
Histamine N-methyltransferase	HNMT	1
Histidine triad nucleotide-binding protein 1	HINT1	2
Histidine triad nucleotide-binding protein 3	HINT3	1
Histidine-rich glycoprotein	HRG	14
Histone H1.5	HIST1H1B	4
Histone H4	HIST1H4A	6
HLA class II histocompatibility antigen, DM alpha chain	HLA-DMA	1
HLA class II histocompatibility antigen, DM beta chain	HLA-DMB	3
HLA class II histocompatibility antigen, DR alpha chain	HLA-DRA	1
Homogentisate 1,2-dioxygenase	HGD	1
Hornerin	HRNR	1
Hsp90 co-chaperone Cdc37	CDC37	1
Hyaluronidase-1	HYAL1	1
Ig alpha-1 chain C region	IGHA1	8
Ig alpha-2 chain C region	IGHA2	1
Ig gamma-2 chain C region	IGHG2	8
Ig heavy chain V-III region NIE	N/A	1
Ig heavy chain V-III region TUR	N/A	1
Ig kappa chain C region	IGKC	1
Ig kappa chain V-III region B6	N/A	2
Ig kappa chain V-III region GOL	N/A	2
Ig kappa chain V-III region HAH	N/A	2

Protein name	Gene name	Number of unique peptides (seq, z)
Ig kappa chain V-III region NG9 (Fragment)	N/A	1
Ig kappa chain V-III region SIE	N/A	3
Ig kappa chain V-III region Ti	N/A	2
Ig kappa chain V-III region WOL	N/A	3
Ig kappa chain V-IV region B17	N/A	2
Ig kappa chain V-IV region JI	N/A	1
Ig kappa chain V-IV region Len	N/A	2
Ig lambda chain V-I region HA	N/A	1
Ig lambda chain V-I region NEWM	N/A	2
Ig lambda chain V-I region WAH	N/A	1
Ig lambda chain V-III region LOI	N/A	1
Ig mu chain C region	IGHM	1
IgGFc-binding protein	FCGBP	2
IgGFc-binding protein	FCGBP	18
Immunoglobulin heavy constant alpha 2 (Fragment)	IGHA2	1
Immunoglobulin heavy constant gamma 3	IGHG3	4
Immunoglobulin heavy constant gamma 4	IGHG4	6
Immunoglobulin heavy constant mu (Fragment)	IGHM	1
Immunoglobulin heavy variable 1-18	IGHV1-18	1
Immunoglobulin heavy variable 3-49	IGHV3-49	1
Immunoglobulin heavy variable 5-51	IGHV5-51	1
Immunoglobulin J chain	JCHAIN	6
Immunoglobulin kappa variable 2D-24 (non-functional) (Fragment)	IGKV2D-24	1
Immunoglobulin kappa variable 3-11	IGKV3-11	1
Immunoglobulin kappa variable 3D-20	IGKV3D-20	1
Immunoglobulin kappa variable 4-1	IGKV4-1	2
Immunoglobulin lambda-like polypeptide 1	IGLL1	1
Immunoglobulin superfamily containing leucine-rich repeat protein	ISLR	4
Immunoglobulin superfamily member 8	IGSF8	4
Importin subunit beta-1	KPNB1	2
Inactive phospholipase D5	PLD5	1
InaD-like protein	INADL	10
Inorganic pyrophosphatase 2, mitochondrial	PPA2	1
Insulin-like growth factor-binding protein 2	IGFBP2	8
Insulin-like growth factor-binding protein 7	IGFBP7	5
Insulin-like growth factor-binding protein complex acid labile subunit	IGFALS	3
Integral membrane protein 2B	ITM2B	5
Integrin alpha-3	ITGA3	2
Integrin alpha-V	ITGAV	10
Integrin beta-1	ITGB1	8
Integrin beta-2	ITGB2	1

Protein name	Gene name	Number of unique peptides (seq, z)
Integrin beta-3	ITGB3	1
Integrin beta-5	ITGB5	3
Inter-alpha-trypsin inhibitor heavy chain H1	ITIH1	4
Inter-alpha-trypsin inhibitor heavy chain H2	ITIH2	11
Inter-alpha-trypsin inhibitor heavy chain H4	ITIH4	23
Interleukin-1 receptor antagonist protein	IL1RN	1
Isocitrate dehydrogenase [NADP] cytoplasmic	IDH1	13
IST1 homolog (Fragment)	IST1	11
IST1 homolog (Fragment)	IST1	3
IST1 homolog	IST1	18
ITIH4 protein	ITIH4	2
Junction plakoglobin	JUP	7
Junctional adhesion molecule A	F11R	1
Kallistatin	SERPINA4	3
Kelch domain-containing protein 7B	KLHDC7B	1
Keratin, type I cuticular Ha1	KRT31	1
Keratin, type I cuticular Ha3-II	KRT33B	1
Keratin, type I cytoskeletal 10	KRT10	39
Keratin, type I cytoskeletal 13	KRT13	3
Keratin, type I cytoskeletal 14	KRT14	13
Keratin, type I cytoskeletal 16	KRT16	14
Keratin, type I cytoskeletal 17	KRT17	5
Keratin, type I cytoskeletal 18	KRT18	1
Keratin, type I cytoskeletal 19	KRT19	3
Keratin, type I cytoskeletal 23	KRT23	2
Keratin, type I cytoskeletal 9	KRT9	40
Keratin, type II cuticular Hb2	KRT82	1
Keratin, type II cuticular Hb4	KRT84	2
Keratin, type II cuticular Hb5	KRT85	2
Keratin, type II cytoskeletal 1	KRT1	56
Keratin, type II cytoskeletal 1b	KRT77	7
Keratin, type II cytoskeletal 2 epidermal	KRT2	43
Keratin, type II cytoskeletal 3	KRT3	1
Keratin, type II cytoskeletal 4	KRT4	3
Keratin, type II cytoskeletal 5	KRT5	18
Keratin, type II cytoskeletal 6A	KRT6A	2
Keratin, type II cytoskeletal 6B	KRT6B	2
Keratin, type II cytoskeletal 7	KRT7	7
Keratin, type II cytoskeletal 74	KRT74	1
Keratin, type II cytoskeletal 78	KRT78	3
Keratin, type II cytoskeletal 8	KRT8	6
Keratin, type II cytoskeletal 80	KRT80	2
Keratin-associated protein 3-1	KRTAP3-1	1
Keratinocyte proline-rich protein	KPRP	8

Protein name	Gene name	Number of unique peptides (seq, z)
Kin of IRRE-like protein 1	KIRREL	1
Kin of IRRE-like protein 3	KIRREL3	1
Kinesin-1 heavy chain	KIF5B	1
Kinesin-like protein KIF12	KIF12	17
Kininogen-1	KNG1	17
Kunitz-type protease inhibitor 2	SPINT2	2
Lactotransferrin	LTF	11
Lactoylglutathione lyase	GLO1	5
Lambda-crystallin homolog	CRYL1	3
Laminin subunit alpha-2	LAMA2	1
LanC-like protein 1	LANCL1	2
Leucine-rich alpha-2-glycoprotein	LRG1	23
Leucine-rich repeat-containing protein 57	LRRC57	2
Leucine-rich repeat-containing protein 71	LRRC71	1
Leukocyte elastase inhibitor	SERPINB1	2
Leukocyte surface antigen CD47	CD47	1
Leukotriene A-4 hydrolase	LTA4H	1
LIM and SH3 domain protein 1	LASP1	4
LIM domain and actin-binding protein 1	LIMA1	1
Lipopolysaccharide-binding protein	LBP	1
Lipopolysaccharide-induced tumor necrosis factor-alpha factor	LITAF	1
LisH domain-containing protein ARMC9	ARMC9	1
L-lactate dehydrogenase A chain	LDHA	3
L-lactate dehydrogenase B chain	LDHB	14
Long-chain fatty acid transport protein 4	SLC27A4	1
Long-chain-fatty-acid--CoA ligase 4	ACSL4	3
Loricrin	LOR	1
Low molecular weight phosphotyrosine protein phosphatase	ACP1	3
Low-density lipoprotein receptor-related protein 2	LRP2	25
Lumican	LUM	5
L-xylulose reductase	DCXR	1
Ly6/PLAUR domain-containing protein 2	LYPD2	1
Lymphocyte antigen 6D	LY6D	2
Lysine-specific demethylase 2B	KDM2B	1
Lysophosphatidylcholine acyltransferase 2	LPCAT2	1
Lysosomal acid phosphatase	ACP2	5
Lysosomal alpha-glucosidase	GAA	8
Lysosome-associated membrane glycoprotein 1	LAMP1	4
Lysosome-associated membrane glycoprotein 2	LAMP2	3
Macrophage migration inhibitory factor	MIF	2
Macrophage-capping protein	CAPG	5
MAGUK p55 subfamily member 5	MPP5	10

Protein name	Gene name	Number of unique peptides (seq, z)
Malate dehydrogenase, cytoplasmic	MDH1	10
Malate dehydrogenase, mitochondrial	MDH2	2
Maltase-glucoamylase, intestinal	MGAM	6
Maltase-glucoamylase, intestinal	MGAM	8
Mannan-binding lectin serine protease 2	MASP2	10
Mannose-6-phosphate isomerase	MPI	1
Mannosyl-oligosaccharide 1,2-alpha-mannosidase IA	MAN1A1	6
MARCKS-related protein	MARCKSL1	1
Matrix metalloproteinase-9	MMP9	1
Matrix-remodeling-associated protein 8	MXRA8	12
Megakaryocyte-associated tyrosine-protein kinase	MATK	1
Melanotransferrin	MELTF	9
Merlin	NF2	3
Methylthioribose-1-phosphate isomerase	MRI1	1
Mirror-image polydactyly gene 1 protein	MIPOL1	1
MIT domain-containing protein 1	MITD1	6
Mitoguardin 1	MIGA1	1
Moesin	MSN	32
Monocyte differentiation antigen CD14	CD14	17
Mucin-1	MUC1	2
Mucin-1	MUC1	7
Mucin-1	MUC1	11
Mucin-13	MUC13	2
Mucin-21	MUC21	1
Mucin-4	MUC4	11
Mucin-4	MUC4	12
Mucin-5AC (Fragments)	MUC5AC	24
Mucin-5B	MUC5B	50
Mucin-6	MUC6	1
Mucin-6	MUC6	17
Mucin-like protein 1	MUCL1	1
Mucosal addressin cell adhesion molecule 1	MADCAM1	1
Multidrug resistance protein 1	ABCB1	18
Multifunctional protein ADE2	PAICS	2
Multimerin-2	MMRN2	2
Multiple epidermal growth factor-like domains protein 8	MEGF8	5
Multivesicular body subunit 12A	MVB12A	8
Multivesicular body subunit 12B	MVB12B	1
Myeloblastin	PRTN3	2
Myeloperoxidase	MPO	9
Myocilin	MYOC	1
Myoferlin	MYOF	7
Myoglobin	MB	1
Myosin light polypeptide 6	MYL6	2

Protein name	Gene name	Number of unique peptides (seq, z)
Myosin-9	MYH9	11
Myotubularin-related protein 14	MTMR14	1
Myristoylated alanine-rich C-kinase substrate	MARCKS	2
N(G),N(G)-dimethylarginine dimethylaminohydrolase 1	DDAH1	2
N(G),N(G)-dimethylarginine dimethylaminohydrolase 2	DDAH2	11
Na(+)/H(+) exchange regulatory cofactor NHE-RF1	SLC9A3R1	11
Na(+)/H(+) exchange regulatory cofactor NHE-RF2	SLC9A3R2	6
Na(+)/H(+) exchange regulatory cofactor NHE-RF3	PDZK1	5
N-acetyl-D-glucosamine kinase	NAGK	1
N-acetylgalactosamine-6-sulfatase	GALNS	3
N-acetylglucosamine-6-sulfatase	GNS	4
N-acetylmuramoyl-L-alanine amidase	PGLYRP2	14
NADH dehydrogenase [ubiquinone] iron-sulfur protein 5	NDUFS5	1
Napsin-A	NAPSA	5
Nck-associated protein 1	NCKAP1	1
Nectin-2 (Fragment)	NECTIN2	2
Nectin-2	NECTIN2	7
Nectin-4	NECTIN4	3
NEDD4-like E3 ubiquitin-protein ligase WWP1	WWP1	1
NEDD8	NEDD8	1
Nephrin	NPHS1	4
Neprilysin	MME	28
Neural Wiskott-Aldrich syndrome protein	WASL	3
Neuroblast differentiation-associated protein AHNAK	AHNAK	12
Neurofascin	NFASC	1
Neurofibromin	NF1	1
Neuroligin-3	NLGN3	1
Neurosecretory protein VGF	VGF	1
Neutral and basic amino acid transport protein rBAT	SLC3A1	1
Neutrophil elastase	ELANE	3
Neutrophil gelatinase-associated lipocalin	LCN2	2
NF-X1-type zinc finger protein NFXL1	NFXL1	1
Niban-like protein 1	FAM129B	4
Nicastrin	NCSTN	1
Nicotinate phosphoribosyltransferase	NAPRT	3
Nicotinate-nucleotide pyrophosphorylase [carboxylating]	QPRT	4
Nidogen-1	NID1	9
Nitric oxide synthase, inducible	NOS2	1
Nitrilase homolog 1	NIT1	1
Non-secretory ribonuclease	RNASE2	1
Nuclear transport factor 2	NUTF2	2
Nucleobindin-1	NUCB1	3
Nucleolar pre-ribosomal-associated protein 1	URB1	1
Nucleoside diphosphate kinase B	NME2	1

Protein name	Gene name	Number of unique peptides (seq, z)
Nucleoside diphosphate kinase	NME1-NME2	1
Olfactomedin-4	OLFM4	19
Omega-amidase NIT2	NIT2	2
Osteoclast-associated immunoglobulin-like receptor	OSCAR	4
Osteoclast-associated immunoglobulin-like receptor	OSCAR	4
Osteoclast-stimulating factor 1	OSTF1	2
Oxidoreductase NAD-binding domain-containing protein 1	OXNAD1	1
Pancreatic alpha-amylase	AMY2A	4
Pantetheinase	VNN1	1
Parathyroid hormone/parathyroid hormone-related peptide receptor	PTH1R	1
Partitioning defective 3 homolog B	PARD3B	1
Partitioning defective 6 homolog beta	PARD6B	2
PDZ domain-containing protein GIPC1	GIPC1	4
Peflin	PEF1	5
Pendrin	SLC26A4	1
Peptidase inhibitor 16	PI16	1
Peptidoglycan recognition protein 1	PGLYRP1	7
Peptidyl-glycine alpha-amidating monooxygenase	PAM	1
Peptidyl-prolyl cis-trans isomerase A	PPIA	17
Peptidyl-prolyl cis-trans isomerase B	PPIB	1
Peptidyl-prolyl cis-trans isomerase FKBP1A	FKBP1A	1
Periplakin	PPL	29
Peroxiredoxin-1	PRDX1	6
Peroxiredoxin-2	PRDX2	13
Peroxiredoxin-5, mitochondrial	PRDX5	2
Peroxiredoxin-6	PRDX6	7
Phosphatase and actin regulator 4	PHACTR4	1
Phosphatidylcholine-sterol acyltransferase	LCAT	3
Phosphatidylethanolamine-binding protein 1	PEBP1	17
Phosphoacetylglucosamine mutase	PGM3	1
Phosphoenolpyruvate carboxykinase, cytosolic [GTP]	PCK1	1
Phosphoglucomutase-1	PGM1	2
Phosphoglycerate kinase 1	PGK1	22
Phosphoglycerate mutase 1	PGAM1	7
Phospholipase D3	PLD3	1
Phospholipid hydroperoxide glutathione peroxidase, mitochondrial	GPX4	1
Phospholipid scramblase 1	PLSCR1	7
Phospholipid scramblase 3	PLSCR3	1
Phospholipid-transporting ATPase IC	ATP8B1	1
Phosphotriesterase-related protein	PTER	2
Pigment epithelium-derived factor	SERPINF1	9

Protein name	Gene name	Number of unique peptides (seq, z)
Pituitary tumor-transforming gene 1 protein-interacting protein	PTTG1IP	2
Plakophilin-1	PKP1	2
Plasma protease C1 inhibitor	SERPING1	7
Plasma serine protease inhibitor	SERPINA5	10
Plasminogen	PLG	16
Plastin-1	PLS1	3
Plastin-2	LCP1	2
Plastin-3	PLS3	6
Platelet glycoprotein Ib alpha chain	GP1BA	1
Platelet-activating factor acetylhydrolase IB subunit beta	PAFAH1B2	1
Platelet-activating factor acetylhydrolase IB subunit gamma	PAFAH1B3	1
Platelet-derived growth factor receptor beta	PDGFRB	5
Pleckstrin homology domain-containing family A member 1	PLEKHA1	1
Pleckstrin homology domain-containing family B member 2	PLEKHB2	1
Pleckstrin homology domain-containing family F member 2	PLEKHF2	1
Plectin	PLEC	1
Plexin-B2	PLXNB2	1
Podocalyxin	PODXL	11
Podocin	NPHS2	4
Poly(rC)-binding protein 1	PCBP1	6
Polycystin-2	PKD2	1
Polymeric immunoglobulin receptor	PIGR	24
Potassium-transporting ATPase alpha chain 2	ATP12A	2
Probable hydrolase PNKD	PNKD	1
Probable serine carboxypeptidase CPVL	CPVL	2
Probable Xaa-Pro aminopeptidase 3	XPNPEP3	1
Procollagen C-endopeptidase enhancer 1	PCOLCE	1
Procollagen-lysine,2-oxoglutarate 5-dioxygenase 3	PLOD3	1
Pro-epidermal growth factor	EGF	24
Profilin	PFN2	2
Profilin-1	PFN1	8
Profilin-2	PFN2	2
Programmed cell death 6-interacting protein	PDCD6IP	55
Programmed cell death protein 10	PDCD10	2
Programmed cell death protein 6	PDCD6	9
Prolactin-inducible protein	PIP	4
Prominin-1	PROM1	32
Prominin-2	PROM2	15
Prosaposin	PSAP	1
Prosaposin	PSAP	4
Prostaglandin reductase 1	PTGR1	9
Prostaglandin-H2 D-isomerase	PTGDS	3
Prostamide/prostaglandin F synthase	FAM213B	2

Protein name	Gene name	Number of unique peptides (seq, z)
Prostasin	PRSS8	1
Prostate stem cell antigen	PSCA	4
Prostate-specific antigen (Fragment)	KLK3	1
Prostate-specific antigen	KLK3	5
Prostatic acid phosphatase	ACPP	12
Proteasome activator complex subunit 1	PSME1	3
Proteasome activator complex subunit 2	PSME2	2
Proteasome subunit alpha type-1	PSMA1	1
Proteasome subunit alpha type-2	PSMA2	1
Proteasome subunit alpha type-5	PSMA5	2
Proteasome subunit alpha type-6	PSMA6	1
Proteasome subunit beta type-2	PSMB2	1
Proteasome subunit beta type-6	PSMB6	1
Protein AMBP	AMBP	15
Protein AMBP	AMBP	1
Protein ARPC4-TTLL3	ARPC4-TTLL3	1
Protein crumbs homolog 2	CRB2	8
Protein crumbs homolog 3	CRB3	1
Protein CutA	CUTA	2
Protein DJ-1	PARK7	7
Protein dopey-2	DOPEY2	1
Protein FAM151A	FAM151A	1
Protein FAM177A1	FAM177A1	1
Protein FAM63A	FAM63A	3
Protein FAM65A	FAM65A	2
Protein KIAA0100	KIAA0100	1
Protein kinase C alpha type	PRKCA	2
Protein kinase C and casein kinase substrate in neurons protein 2	PACSIN2	6
Protein kinase C and casein kinase substrate in neurons protein 3	PACSIN3	11
Protein kinase C zeta type	PRKCZ	1
Protein lifeguard 3	TMBIM1	3
Protein lin-7 homolog C	LIN7C	3
Protein MAL2	MAL2	1
Protein NDRG1	NDRG1	2
Protein phosphatase 1 regulatory subunit 16A	PPP1R16A	1
Protein phosphatase methylesterase 1	PPME1	1
Protein phosphatase Slingshot homolog 1	SSH1	2
Protein S100-A10	S100A10	1
Protein S100-A11	S100A11	6
Protein S100-A12	S100A12	1
Protein S100-A14	S100A14	4
Protein S100-A16	S100A16	4

Protein name	Gene name	Number of unique peptides (seq, z)
Protein S100-A4	S100A4	1
Protein S100-A6	S100A6	1
Protein S100-A7	S100A7	1
Protein S100-A8	S100A8	4
Protein S100-A9	S100A9	10
Protein S100-P	S100P	4
Protein SON	SON	1
Protein stum homolog	C1orf95	1
Protein tweety homolog 3	TTYH3	1
Protein tyrosine phosphatase type IVA 1	PTP4A1	1
Protein XRP2	RP2	2
Protein-glutamine gamma-glutamyltransferase 2	TGM2	2
Protein-glutamine gamma-glutamyltransferase 4	TGM4	8
Protein-glutamine gamma-glutamyltransferase E	TGM3	2
Protein-L-isoaspartate(D-aspartate) O-methyltransferase	PCMT1	1
Prothrombin	F2	11
Protocadherin gamma-B4	PCDHGB4	1
Protocadherin gamma-C3	PCDHGC3	2
Protocadherin-1	PCDH1	2
Protocadherin-12	PCDH12	2
Proto-oncogene tyrosine-protein kinase Src	SRC	2
Purine nucleoside phosphorylase	PNP	1
Puromycin-sensitive aminopeptidase	NPEPPS	3
Putative ankyrin repeat domain-containing protein 31	ANKRD31	1
Putative keratin-87 protein	KRT87P	1
Putative uncharacterized protein encoded by CACTIN-AS1	CACTIN-AS1	1
Pyridoxal kinase	PDXK	3
Pyridoxine-5'-phosphate oxidase	PNPO	1
Pyruvate kinase PKLR	PKLR	1
Pyruvate kinase PKM	PKM	18
Quinone oxidoreductase	CRYZ	5
Rab GDP dissociation inhibitor beta	GDI2	5
Rab11 family-interacting protein 1	RAB11FIP1	1
Rab11 family-interacting protein 4	RAB11FIP4	1
Rab11 family-interacting protein 5	RAB11FIP5	1
Rab11 family-interacting protein 5	RAB11FIP5	1
Radixin	RDX	17
Ragulator complex protein LAMTOR1	LAMTOR1	1
Rap guanine nucleotide exchange factor 3	RAPGEF3	5
Ras GTPase-activating-like protein IQGAP1	IQGAP1	16
Ras-related C3 botulinum toxin substrate 1	RAC1	1
Ras-related protein Rab-10	RAB10	2
Ras-related protein Rab-13	RAB13	1
Ras-related protein Rab-14	RAB14	2

Protein name	Gene name	Number of unique peptides (seq, z)
Ras-related protein Rab-15	RAB15	1
Ras-related protein Rab-17	RAB17	1
Ras-related protein Rab-1A	RAB1A	2
Ras-related protein Rab-21	RAB21	1
Ras-related protein Rab-22A	RAB22A	1
Ras-related protein Rab-25	RAB25	2
Ras-related protein Rab-27A	RAB27A	3
Ras-related protein Rab-27B	RAB27B	2
Ras-related protein Rab-2A	RAB2A	2
Ras-related protein Rab-33B	RAB33B	2
Ras-related protein Rab-35	RAB35	2
Ras-related protein Rab-3B	RAB3B	3
Ras-related protein Rab-3D	RAB3D	2
Ras-related protein Rab-5B	RAB5B	2
Ras-related protein Rab-5C	RAB5C	5
Ras-related protein Rab-7a	RAB7A	11
Ras-related protein Rab-8A	RAB8A	4
Ras-related protein Rab-8B	RAB8B	3
Ras-related protein Ral-A	RALA	3
Ras-related protein Ral-B	RALB	3
Ras-related protein Rap-1A	RAP1A	1
Ras-related protein Rap-2b	RAP2B	1
Ras-related protein Rap-2c	RAP2C	1
Ras-related protein R-Ras	RRAS	4
Ras-related protein R-Ras2	RRAS2	1
Receptor-type tyrosine-protein phosphatase eta	PTPRJ	16
Receptor-type tyrosine-protein phosphatase O	PTPRO	5
Renin receptor	ATP6AP2	2
Resistin	RETN	3
Reticulon-4 receptor-like 2	RTN4RL2	1
Retinal dehydrogenase 1	ALDH1A1	9
Retinoic acid-induced protein 3	GPRC5A	6
Retinol-binding protein 4	RBP4	1
Retinol-binding protein 5	RBP5	1
Rho GDP-dissociation inhibitor 1 (Fragment)	ARHGDIA	2
Rho GDP-dissociation inhibitor 1	ARHGDIA	1
Rho GDP-dissociation inhibitor 1	ARHGDIA	6
Rho GDP-dissociation inhibitor 2	ARHGDIB	1
Rho GTPase-activating protein 18	ARHGAP18	6
Rhophilin-2	RHPN2	1
Rho-related GTP-binding protein RhoB	RHOB	2
Rho-related GTP-binding protein RhoC	RHOC	2
Rho-related GTP-binding protein RhoF	RHOF	5
Rho-related GTP-binding protein RhoG	RHOG	5

Protein name	Gene name	Number of unique peptides (seq, z)
Ribonuclease inhibitor	RNH1	7
Ribonuclease UK114	HRSP12	1
RILP-like protein 2	RILPL2	1
RNA polymerase II elongation factor ELL3	ELL3	1
Roundabout homolog 4	ROBO4	10
Secreted and transmembrane protein 1	SECTM1	3
Secretory carrier-associated membrane protein 2	SCAMP2	1
Secretory phospholipase A2 receptor	PLA2R1	1
Selenium-binding protein 1	SELENBP1	5
Semaphorin-3G	SEMA3G	1
Semaphorin-5A	SEMA5A	3
Semenogelin-1	SEMG1	3
Semenogelin-2	SEMG2	4
Sepiapterin reductase	SPR	2
Septin-2	SEPT2	3
Serine hydroxymethyltransferase, cytosolic	SHMT1	5
Serine incorporator 5	SERINC5	1
Serine protease hepsin	HPN	4
Serine/threonine-protein kinase 24	STK24	8
Serine/threonine-protein kinase 25	STK25	2
Serine/threonine-protein kinase 26	STK26	1
Serine/threonine-protein kinase ATR	ATR	1
Serine/threonine-protein kinase H1	PSKH1	1
Serine/threonine-protein kinase N2	PKN2	1
Serine/threonine-protein kinase PAK 4	PAK4	3
Serine/threonine-protein kinase TAO1	TAOK1	2
Serine/threonine-protein kinase TAO3	TAOK3	2
Serine/threonine-protein kinase ULK3	ULK3	3
Serine/threonine-protein phosphatase 2A 65 kDa regulatory subunit A alpha isoform	PPP2R1A	1
Serine/threonine-protein phosphatase PP1-alpha catalytic subunit	PPP1CA	1
Serine/threonine-protein phosphatase PP1-beta catalytic subunit	PPP1CB	1
Serine--tRNA ligase, cytoplasmic	SARS	1
Serotransferrin	TF	63
Serpin B3	SERPINB3	3
Serpin B6	SERPINB6	1
Serpin B6	SERPINB6	3
Serum albumin	ALB	56
Sex hormone-binding globulin	SHBG	1
S-formylglutathione hydrolase	ESD	4
SH3 and multiple ankyrin repeat domains protein 2	SHANK2	1
SH3 domain-binding glutamic acid-rich-like protein	SH3BGRL	1

Protein name	Gene name	Number of unique peptides (seq, z)
SH3 domain-binding protein 4	SH3BP4	4
Sialate O-acetylesterase	SIAE	1
Sialic acid synthase	NANS	2
Sialidase-1	NEU1	1
Signal transducer and activator of transcription 1-alpha/beta	STAT1	2
Small integral membrane protein 22	SMIM22	2
Small integral membrane protein 24	SMIM24	1
Small integral membrane protein 5	SMIM5	1
Small proline-rich protein 3	SPRR3	10
Small VCP/p97-interacting protein	SVIP	1
Smoothened homolog	SMO	2
Sodium channel protein type 4 subunit alpha	SCN4A	1
Sodium/potassium-transporting ATPase subunit alpha-1	ATP1A1	10
Sodium/potassium-transporting ATPase subunit beta-1	ATP1B1	5
Sodium-dependent neutral amino acid transporter B(0)AT1	SLC6A19	2
Solute carrier family 12 member 1 (Fragment)	SLC12A1	3
Solute carrier family 12 member 1	SLC12A1	26
Solute carrier family 12 member 3	SLC12A3	6
Solute carrier family 2, facilitated glucose transporter member 5	SLC2A5	2
Solute carrier family 22 member 2	SLC22A2	1
Sorbitol dehydrogenase	SORD	9
Sorcin	SRI	10
Sorting and assembly machinery component 50 homolog	SAMM50	1
Sorting nexin-18	SNX18	7
Sorting nexin-33	SNX33	1
SPARC-like protein 1	SPARCL1	1
Spastin	SPAST	1
Specifically androgen-regulated gene protein	SARG	16
Sperm flagellar protein 2	SPEF2	1
Sperm-associated antigen 16 protein	SPAG16	1
SPRY domain-containing SOCS box protein 3	SPSB3	1
Src substrate cortactin	CTTN	2
STAM-binding protein	STAMBP	2
STE20/SPS1-related proline-alanine-rich protein kinase	STK39	1
STE20-like serine/threonine-protein kinase	SLK	9
Stomatin-like protein 3	STOML3	1
Stress-induced-phosphoprotein 1	STIP1	1
Structural maintenance of chromosomes protein 3	SMC3	1
Submaxillary gland androgen-regulated protein 3B	SMR3B	1
Sulfhydryl oxidase 1	QSOX1	2
Superoxide dismutase [Cu-Zn]	SOD1	2
Sushi domain-containing protein 2	SUSD2	2
Synaptic vesicle membrane protein VAT-1 homolog	VAT1	4

Protein name	Gene name	Number of unique peptides (seq, z)
Synaptosomal-associated protein 23	SNAP23	6
Synaptosomal-associated protein 29	SNAP29	1
Synaptotagmin-7	SYT7	1
Synaptotagmin-like protein 1	SYTL1	1
Synaptotagmin-like protein 2	SYTL2	1
Synaptotagmin-like protein 2	SYTL2	4
Synaptotagmin-like protein 4	SYTL4	1
Syndecan binding protein (Syntenin), isoform CRA_a	SDCBP	23
Syntaxin-3	STX3	3
Syntaxin-7	STX7	2
Syntaxin-binding protein 1	STXBP1	2
Syntaxin-binding protein 2	STXBP2	10
Syntaxin-binding protein 4	STXBP4	3
Syntenin-1	SDCBP	24
Syntenin-2	SDCBP2	5
Talin-1	TLN1	3
Tandem C2 domains nuclear protein	TC2N	2
Target of Myb protein 1	TOM1	2
TBC1 domain family member 10A	TBC1D10A	1
TBC1 domain family member 24	TBC1D24	1
T-complex protein 1 subunit beta	CCT2	1
T-complex protein 1 subunit epsilon	CCT5	2
T-complex protein 1 subunit eta	CCT7	1
T-complex protein 1 subunit gamma	CCT3	2
Tenascin-X	TNXB	1
Tenascin-X	TNXB	1
Teneurin-3	TENM3	1
Tetranectin	CLEC3B	1
Tetraspanin (Fragment)	CD9	2
Tetraspanin-1	TSPAN1	4
Tetraspanin-8	TSPAN8	1
Thioredoxin domain-containing protein 17	TXNDC17	2
Thioredoxin	TXN	2
Thioredoxin-like protein 1	TXNL1	1
Thiosulfate sulfurtransferase/rhodanese-like domain-containing protein 1	TSTD1	1
Thrombospondin-3	THBS3	1
Thy-1 membrane glycoprotein	THY1	3
Thymosin beta-4	TMSB4X	1
Thyroxine-binding globulin	SERPINA7	4
Tissue factor	F3	1
Toll-interacting protein	TOLLIP	3
TOM1-like protein 1	TOM1L1	1
TOM1-like protein 2	TOM1L2	4

Protein name	Gene name	Number of unique peptides (seq, z)
Transaldolase	TALDO1	3
Transcription factor AP-2-delta	TFAP2D	1
Transforming growth factor beta receptor type 3	TGFBR3	1
Transforming protein RhoA	RHOA	2
Transgelin-2	TAGLN2	3
Transient receptor potential cation channel subfamily V member 5	TRPV5	1
Transitional endoplasmic reticulum ATPase	VCP	1
Transketolase	TKT	3
Translationally-controlled tumor protein	TPT1	1
Transmembrane 9 superfamily member	N/A	1
Transmembrane channel-like protein 5	TMC5	3
Transmembrane protease serine 11B	TMPRSS11B	1
Transmembrane protease serine 2	TMPRSS2	7
Transmembrane protein 201	TMEM201	1
Transmembrane protein 8A	TMEM8A	1
Transthyretin	TTR	2
Transthyretin	TTR	10
Trefoil factor 2	TFF2	1
Trehalase	TREH	1
Trifunctional purine biosynthetic protein adenosine-3	GART	1
Triokinase/FMN cyclase	TKFC	5
Triosephosphate isomerase	TPI1	23
Tripartite motif-containing protein 47	TRIM47	1
Tripeptidyl-peptidase 1	TPP1	7
Tropomyosin alpha-4 chain	TPM4	1
Troponin T, slow skeletal muscle	TNNT1	1
Tryptophan--tRNA ligase, cytoplasmic	WARS	1
Tubulin alpha-4A chain	TUBA4A	2
Tubulin beta chain	TUBB	1
Tubulin beta-4A chain	TUBB4A	1
Tubulin beta-4B chain	TUBB4B	2
Tubulin polymerization-promoting protein family member 3	TPPP3	1
Tumor necrosis factor alpha-induced protein 2	TNFAIP2	2
Tumor necrosis factor receptor superfamily member 19L	RELT	1
Tumor susceptibility gene 101 protein	TSG101	16
Tumor-associated calcium signal transducer 2	TACSTD2	2
Type I inositol 1,4,5-trisphosphate 5-phosphatase	INPP5A	1
Tyrosine-protein kinase FRK	FRK	14
Tyrosine-protein kinase Lyn	LYN	5
Tyrosine-protein kinase	YES1	1
Tyrosine-protein kinase receptor UFO	AXL	2
Tyrosine-protein kinase Yes	YES1	6

Protein name	Gene name	Number of unique peptides (seq, z)
Tyrosine-protein phosphatase non-receptor type 13	PTPN13	16
Tyrosine-protein phosphatase non-receptor type 6	PTPN6	1
Ubiquitin carboxyl-terminal hydrolase 5	USP5	1
Ubiquitin carboxyl-terminal hydrolase isozyme L1	UCHL1	4
Ubiquitin carboxyl-terminal hydrolase isozyme L3	UCHL3	1
Ubiquitin carboxyl-terminal hydrolase	UCHL1	1
Ubiquitin domain-containing protein 1	UBTD1	1
Ubiquitin-conjugating enzyme E2 variant 3	UEVLD	4
Ubiquitin-fold modifier-conjugating enzyme 1	UFC1	1
Ubiquitin-like modifier-activating enzyme 1	UBA1	14
Ubiquitin-like protein 3	UBL3	1
UBX domain-containing protein 6	UBXN6	3
UMP-CMP kinase	CMPK1	5
Uncharacterized protein C11orf52	C11orf52	7
Uncharacterized protein C4orf19	C4orf19	1
Uncharacterized protein C6orf132	C6orf132	1
Uncharacterized protein C9orf66	C9orf66	1
Uncharacterized protein KIAA1522	KIAA1522	2
Unconventional myosin-Ib	MYO1B	11
Unconventional myosin-Ic	MYO1C	46
Unconventional myosin-Id	MYO1D	10
Unconventional myosin-Ie	MYO1E	1
Unconventional myosin-VI	MYO6	4
Unconventional myosin-VI	MYO6	7
Urokinase-type plasminogen activator	PLAU	1
Uromodulin (Fragment)	UMOD	1
Uromodulin	UMOD	40
Uroplakin-1a	UPK1A	9
Uroplakin-1b	UPK1B	1
Uroplakin-2	UPK2	5
Uroplakin-3a	UPK3A	7
Uroplakin-3b	UPK3B	1
USP6 N-terminal-like protein	USP6NL	1
USP6 N-terminal-like protein	USP6NL	1
UTP--glucose-1-phosphate uridylyltransferase	UGP2	1
UV excision repair protein RAD23 homolog B	RAD23B	1
Uveal autoantigen with coiled-coil domains and ankyrin repeats	UACA	1
Vacuolar protein sorting-associated protein 28 homolog	VPS28	4
Vacuolar protein sorting-associated protein 29	VPS29	1
Vacuolar protein sorting-associated protein 35	VPS35	1
Vacuolar protein sorting-associated protein 37B	VPS37B	7
Vacuolar protein sorting-associated protein 37C	VPS37C	1
Vacuolar protein sorting-associated protein 37D	VPS37D	11

Protein name	Gene name	Number of unique peptides (seq, z)
Vacuolar protein sorting-associated protein 4A	VPS4A	13
Vacuolar protein sorting-associated protein 4B	VPS4B	17
Vacuolar protein sorting-associated protein VTA1 homolog	VTA1	5
Vacuolar protein-sorting-associated protein 25	VPS25	3
Vacuolar protein-sorting-associated protein 36	VPS36	2
Vascular cell adhesion protein 1	VCAM1	1
Vasorin	VASN	20
Versican core protein (Fragment)	VCAN	7
Versican core protein	VCAN	7
Vesicle-fusing ATPase	NSF	2
Vesicular integral-membrane protein VIP36	LMAN2	13
Villin-1	VIL1	4
Villin-like protein	VILL	1
Vinculin	VCL	5
Vitamin D-binding protein	GC	1
Vitamin D-binding protein	GC	5
Vitamin K-dependent protein Z	PROZ	2
Vitelline membrane outer layer protein 1 homolog	VMO1	3
Vitronectin	VTN	5
Voltage-dependent anion-selective channel protein 1	VDAC1	4
Voltage-dependent anion-selective channel protein 3	VDAC3	1
Voltage-dependent calcium channel subunit alpha-2/delta-1	CACNA2D1	5
VPS9 domain-containing protein 1	VPS9D1	1
V-set domain-containing T-cell activation inhibitor 1	VTCN1	1
V-type proton ATPase 116 kDa subunit a isoform 4	ATP6V0A4	2
V-type proton ATPase catalytic subunit A	ATP6V1A	16
V-type proton ATPase subunit B, brain isoform	ATP6V1B2	2
V-type proton ATPase subunit B, kidney isoform	ATP6V1B1	6
V-type proton ATPase subunit d 1	ATP6V0D1	2
V-type proton ATPase subunit E 1	ATP6V1E1	3
V-type proton ATPase subunit G 1	ATP6V1G1	1
V-type proton ATPase subunit H	ATP6V1H	3
WAS/WASL-interacting protein family member 2	WIPF2	1
WD repeat-containing protein 1	WDR1	10
Wiskott-Aldrich syndrome protein family member 2	WASF2	3
Xaa-Pro aminopeptidase 1	XPNPEP1	1
Xaa-Pro aminopeptidase 2	XPNPEP2	9
Xaa-Pro dipeptidase	PEPD	2
Xin actin-binding repeat-containing protein 1	XIRP1	2
Zinc finger CCCH-type antiviral protein 1	ZC3HAV1	1
Zinc finger protein 185	ZNF185	1
Zinc-alpha-2-glycoprotein	AZGP1	13
